# Microwave Assisted Synthesis of Porous NiCo_2_O_4_ Microspheres: Application as High Performance Asymmetric and Symmetric Supercapacitors with Large Areal Capacitance

**DOI:** 10.1038/srep22699

**Published:** 2016-03-03

**Authors:** Syed Khalid, Chuanbao Cao, Lin Wang, Youqi Zhu

**Affiliations:** 1 Research Center of Materials Science, Beijing Institute of Technology, Beijing 100081, P. R. China

## Abstract

Large areal capacitance is essentially required to integrate the energy storage devices at the microscale electronic appliances. Energy storage devices based on metal oxides are mostly fabricated with low mass loading per unit area which demonstrated low areal capacitance. It is still a challenge to fabricate supercapacitor devices of porous metal oxides with large areal capacitance. Herein we report microwave method followed by a pyrolysis of the as-prepared precursor is used to synthesize porous nickel cobaltite microspheres. Porous NiCo_2_O_4_ microspheres are capable to deliver large areal capacitance due to their high specific surface area and small crystallite size. The facile strategy is successfully demonstrated to fabricate aqueous-based asymmetric & symmetric supercapacitor devices of porous NiCo_2_O_4_ microspheres with high mass loading of electroactive materials. The asymmetric & symmetric devices exhibit maximum areal capacitance and energy density of 380 mF cm^−2^ & 19.1 Wh Kg^−1^ and 194 mF cm^−2^ & 4.5 Wh Kg^−1^ (based on total mass loading of 6.25 & 6.0 mg) respectively at current density of 1 mA cm^−2^. The successful fabrication of symmetric device also indicates that NiCo_2_O_4_ can also be used as the negative electrode material for futuristic asymmetric devices.

Supercapacitors have recently received much attention due to its extremely rapid delivery of stored energy owing to its faster charge-discharge rate and longer life span than batteries^1^. Supercapacitors are extremely suitable for application requiring high power density such as portable electronic devices, starting power devices for fuel cells, space flight technology, hybrid vehicles, renewable energy systems and so on^1^,^2^. Recently the research has been focused intensively to develop supercapacitor devices with large areal capacitance to meet the demand of miniaturization of consumer electronics, hybrid electric vehicles and micro energy systems^3^. Electrochemical capacitors (ECs) are classified as electrical double layer capacitors (EDLCs) or pseudocapacitors based on the electrode material and charge storage mechanism^2^. Currently the carbon based symmetric EDLCs are being used as the supercapacitors in commercial applications^1^. EDLCs usually have energy density of 3–4 Wh kg^−1^ and power density of 3–4 KW kg^−1^ in both aqueous and organic electrolyte^4^. Major challenge is to enhance the energy density of EDLCs so that their usage in the application requiring high power sources with sustainable energy density can be realized^1^. It is also highly desirable to attain the large areal capacitance to design the energy storage devices for miniaturized electronic systems^5^. Large areal capacitance is also an indicative of the material ability to store more energy per unit area.

Energy density of supercapacitor can be enhanced by increasing the capacitance and/or operating voltage window according to formulae 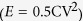. The use of redox active materials enhance substantially the energy density of supercapacitors due to their much higher specific capacitance 10–100 times more than carbonaceous materials^2^. In case of aqueous-based supercapacitor devices, the voltage window of symmetric supercapacitor (SSC) is normally restricted up to 1.0 V but for asymmetric supercapacitor (ASC) it can be extended up to 2 V by using redox-active material cathode and activated carbon anode^6^. The active carbon as anode and redox active material as cathode provide the high hydrogen and oxygen over potential respectively which enhances the potential window of aqueous-based ASC^6^. At present Ruthenium oxide (RuO_2_) is being mostly used as the cathode material of ECs but it is well known that the RuO_2_ is the most expensive and toxic rare metal oxide. The development of environmental benign and cheaper redox active materials (such as hydroxides/oxides of Mn, Co, Ni, Fe, Cr, Mo) with unique structures are of great importance to replace RuO_2_. Few reports have been published on the development of supercapacitor based on oxides of Mo and Mn due to their low cost and environmental benignity^7^,^8^,^9^. The low electrical conductivity of Mo and its structural collapse during cycling process in the aqueous electrolyte have been handled successfully by making its nanocomposite with polypyrrole (PPy)^7^. The rate and cycling performance of MnO_2_ based supercapacitors have been enhanced by preparing the crystalline NaMnO_2_ and K_0.27_ MnO_2_·0.6H_2_O as electrode material^8^,^9^. The binary metal oxides like spinel cobaltites (MCo_2_O_4_; M = Mn, Ni, Zn, etc) offer more oxidation states for Faradaic redox reaction due to contribution from both Co and M ions^10^. Among these transition metal oxides NiCo_2_O_4_ has been considered as the most promising energy storage material due to its higher electrical conductivity by 2 orders of magnitude than the monometallic oxides of Ni or Co^11^. Additionally it is also considered as the most cost effective and scalable material due to its low cost, abundant resources and environmental friendliness^2^. The various nanostructures of NiCo_2_O_4_ such as nanowires^12^, nanosheets^13^, and urchin-like structure^10^, have been explored and demonstrated distinct differences in their electrochemical performance. Recently the development of hierarchically three-dimensional (3D) metal oxides structures have been considered much attention due to their fascinating applications in many fields such as batteries^14^, catalysis^15^, and supercapacitors^10^. The various methods such as sol-aero-gel^16^, chemical bath precipitation^17^, and thermal decomposition^18^, have been used to prepare porous microstructures of metal oxides. Although the above methods have already demonstrated the successful preparation of porous structures but these methods are not users friendly and also involved complicated fabrication processes, high energy consumption and toxic chemical agents. In order to scale up the mass production, it is also important to consider the simplicity and practicability of synthesis methods. Herein we report microwave method to synthesize porous nanostructures of NiCo_2_O_4_ microspheres with simple post calcination process. This technique is considered as the most economical and environmental friendly due to its reduced energy consumption, very rapid heating rates, considerably reduced synthesis time and direct interaction of electromagnetic waves with the reactants instead of indirect heat transfer of conventional methods^19^,^20^,^21^,^22^,^23^,^24^. It is also important to mention that most of the reported research work on these transition metal oxides/hydroxides based on three electrode configuration^13^,^19^,^25^,^26^. The real device which exhibits the practical application of supercapacitor is two electrode system rather than three electrode configuration. So it is also very important to harness the fabrication technology of two electrode system, so that potential of these transition metal oxides/hydroxides based supercapacitors can be used for practical applications. The other important aspect of our work is the fabrication of asymmetric & symmetric supercapacitor devices with high mass loadings on both positive and negative electrodes to attain optimum areal capacitance. Porous NiCo_2_O_4_ microspheres are capable to deliver large areal capacitance due to their high surface area, large pore volume and small crystallite size which maximizes its utility with high mass loading. The total mass loading of active materials on both electrodes of asymmetric & symmetric supercapacitor devices were maintained at 6.25 & 6.0 mg respectively. It is rather difficult to fabricate the aqueous based symmetric devices because for its successful operation both electrodes should have equal thickness, size and mass loading. But it is also quite significant to develop symmetric devices because it can also reduce the dependence on carbon materials. Carbon materials, particularly with high surface area are expensive (US$50–100 per kg)^27^. Secondly the carbon materials with high surface area are produced with harsh processing condition. The successful fabrication of symmetric device also indicates that the porous NiCo_2_O_4_ microspheres can also be used as the negative electrode material in place of active carbon in the futuristic asymmetric devices. It can prove more useful as the negative electrode material for asymmetric devices because porous NiCo_2_O_4_ microspheres have higher specific capacitance than active carbon. Both aqueous based devices have few advantages and disadvantages which should be considered for their specific practical applications. Symmetric devices have shorter charge-discharge time and more safer in comparison with the asymmetric devices and batteries due to its lower potential window. Symmetric devices also exhibit high power densities, long cycle life compared to batteries and comparable energy density to conventional dielectric capacitors. Symmetric devices have no polarity due to same material as an anode and cathode which also prevent the catastrophic failure of device. Whereas asymmetric devices are inherently polar, operate within a large potential window and exhibit battery-like behaviour in providing high energy density. Asymmetric devices require robust encapsulation to prevent the leakage of electrolyte which can occur due to its large potential window. Asymmetric devices are considered as intermediate between conventional dielectric capacitors and batteries. As-synthesized material exhibit outstanding electrochemical performance as an electrode material of ASC & SSC owing to its highly porous structure with average crystallite size around 8 nm, distinct uniform microspheres morphology, relatively high specific surface area, large pore volume and narrow pore size distribution.

## Results

### Structural and morphological characterizations

**Figure 1:**
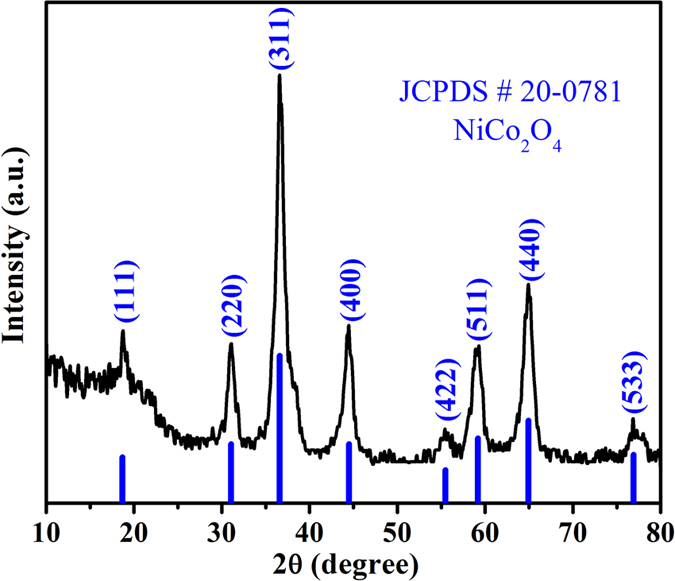
XRD pattern of as-synthesized porous NiCo_2_O_4_ microspheres.

As-prepared precursor was initially characterized by X-ray diffraction (XRD) and Thermogravimetric analysis (TGA) to identify the phases and to determine the follow up calcination temperature (Figs S1 and S2) respectively. TGA study reveals that 300 °C is appropriate temperature for the calcination of as-prepared precursor to form pure phase of NiCo_2_O_4_. The calcinated powder was further characterized by XRD to identify the purity, phase and average crystallite size (Fig. 1). All peaks are well indexed to NiCo_2_O_4_ cubic phase (JCPDS card no. 20–0781). The absence of any unidentified peak in the XRD pattern also indicates the purity of calcinated powder. The presence of broad diffraction peaks also indicate the nanocrystalline nature of as-synthesized material. XRD result also confirms that 300 °C is the appropriate temperature for thermal conversion of as-prepared precursor to NiCo_2_O_4_ powder as indicated by TGA. The average crystallite size of as-synthesized porous NiCo_2_O_4_ was calculated by using Debye-Scherer’s formula given in equation (1).


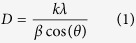


where D(nm) is the crystallite size, K(0.9), λ(*0.15406* nm) is the K_α_ component of wavelength of Cu radiation source, β is the full width at half maximum of individual observed peak and Θ is the Bragg’s angle.

Table S1 summarizes the crystallite size of porous NiCo_2_O_4_ microspheres along different orientations. The average crystallite size around 8.0 nm indicates the nanocrystalline nature of as-synthesized material. The small crystallite size of active material will also enhance its maximum utility during surface chemical reaction. Energy dispersive X-ray spectroscopy (EDX), Fourier transform infrared spectroscopy (FTIR) and X-ray photoelectron spectroscopy (XPS) were carried out to determine the elemental composition, characteristic bands and surface oxidation states of the as-synthesized material (Figs S3–S5). The results of XRD, FTIR, EDX and XPS analysis demonstrate advantages of the microwave method in producing high-purity samples. We performed the detail study of N_2_ adsorption-desorption isotherm of the porous NiCo_2_O_4_ microspheres to determine the specific surface area, pore size distribution and pore volume (Fig. S6). BET measurements indicated high specific surface area (119.68 m^2^ g^−1^), large pore volume (0.4664 cm^3^ g^−1^), mesoporous porosity and narrow pore size distribution mainly centres at 8.7 nm (Fig. S6). The high specific surface area will increase the contact area at electrolyte/electrode interface which will provide abundant active sites for Faradaic reaction during electrochemical reaction. The large pore volume can serve as a reservoir for ions and also greatly enhance the diffusion kinetics within the electrode material. The narrow and ordered distribution of pores which centres at 8.7 nm corresponds to optimum pore size for excellent electrochemical application^12^,^13^. It will provide more facile penetration of electrolyte within the pores for fast Faradaic redox reaction and double layer charging-discharging process. The mesoporous porosity as indicated by the pore size distribution will provide the fast diffusion of ions and electron to the electrode material.

**Figure 2:**
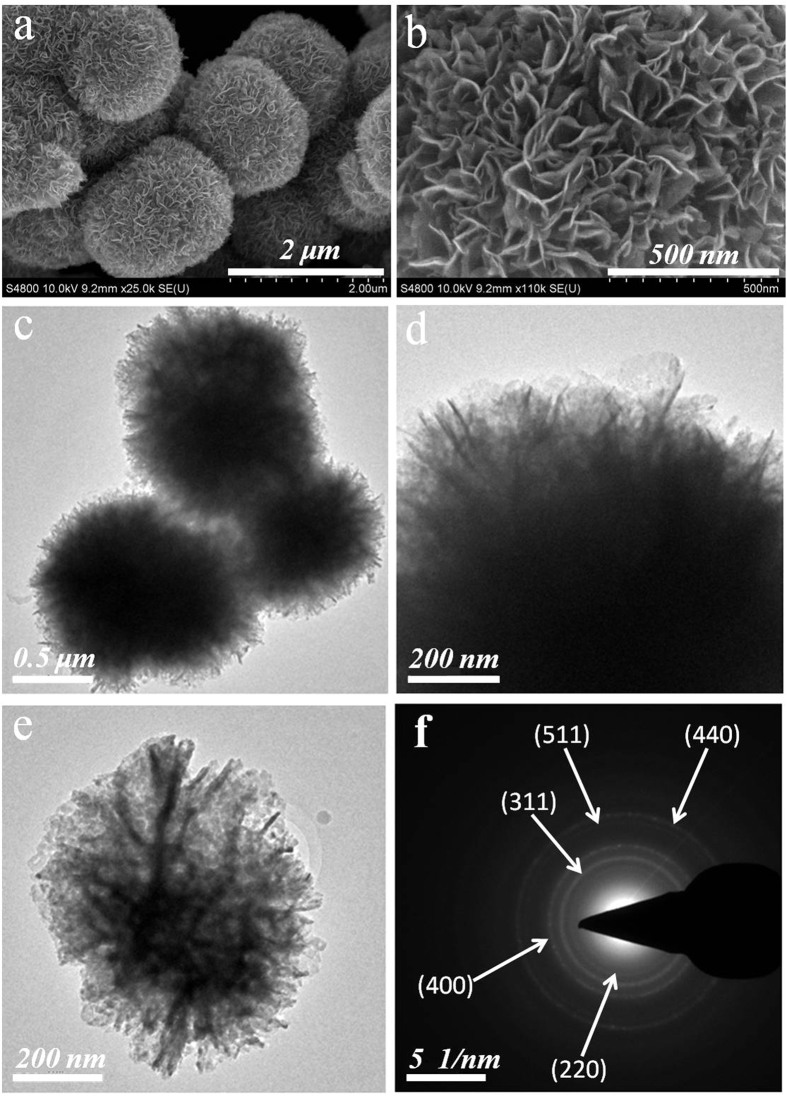
(**a,b**) FE-SEM, (**c–e**) TEM images at different magnifications, (**f**) SAED pattern of porous NiCo_2_O_4_ microspheres.

In order to explore the effect of microwave on nanostructures of NiCo_2_O_4_ time dependent synthesis (10 to 40 minutes) were carried out. Scanning electron microscopy (SEM) was used to harness the morphology and growth mechanism of microspheres (Figs 2a,b and S7). SEM images indicate that the as-synthesized material consists of porous microspheres. Further it is also evident from SEM images that the numerous randomly distributed interconnecting thin nanosheets are assembled together to form each microsphere. The most importantly sufficient void spaces in and between the microspheres can also be clearly seen (Figs 2a,b and S7). SEM was used to optimize the synthesis time which delivers high product yield with fully developed surface morphology and more uniform size distribution of microspheres (Fig. S7). SEM images also indicate that the fully developed microspheres which constituted with enhanced number density of nanosheets with more uniform size distribution are formed at the synthesize duration of 30 minutes (Fig. S7). The detailed interior microstructure and crystallographic properties of these microspheres were further explored by using Transmission electron microscopy (TEM) and Selected area electron diffraction (SAED). TEM images (Fig. 2c,d) indicate that the as-synthesized material have a spherical morphology and the edge of microsphere display its loose packed structure made from many nanosheets that reach far from interior which is consistent with the SEM findings. It is also obvious from the high magnification TEM image (Fig. 2e) that the blank area in the particles shows that these nanosheets have porous structure on their surface. Further the interconnection of nanoparticles can be clearly observed (Fig. 2e) which is also an indicative of formation of mesoporous structure. These mesopores are formed as a result of thermal decomposition of hydroxides and subsequent recrystallization at a relatively low temperature. SAED patterns of NiCo_2_O_4_ (Fig. 2f) further reveals that as-synthesized material has polycrystalline nature with well defined rings. All the observed rings (Fig. 2f) are well indexed with characteristics planes of NiCo_2_O_4_ which is in accordance with the XRD results. The mesoporous porosity as indicated by TEM & pore size distribution, sufficient void spaces in and between the microspheres as revealed by SEM, and polycrystalline nature as indicated by SAED will provide numerous electroactive sites for the fast transport of electrolyte to electrode material during electrochemical reaction.

### Electrochemical analysis

**Figure 3:**
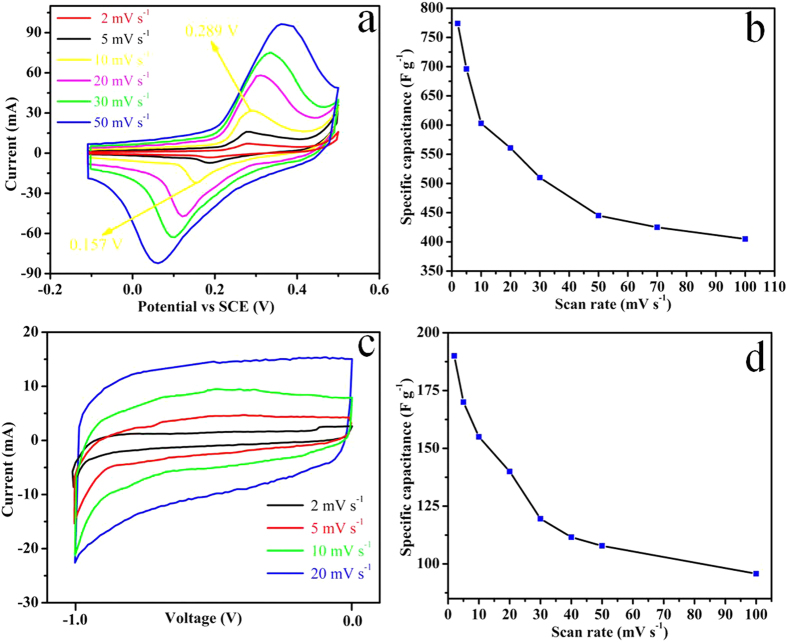
(**a,b**) CV curves and specific capacitances of porous NiCo_2_O_4_ microspheres, (**c,d**) CV curves and specific capacitances of active carbon at various scan rates.

Cyclic voltammetry (CV) was used to determine the capacitive performance of porous NiCo_2_O_4_ microspheres and active carbon in standard three electrode cell configuration. CV curves of porous NiCo_2_O_4_ microspheres at different scan rate ranging from 2 to 100 mV s^−1^ within the potential window −0.1 to 0.5 V vs saturated calomel electrode (Figs 3a and S8). The well defined redox peaks are observed during cathodic and anodic sweep which represents the pseudocapacitive characteristics of as-synthesized material^12^,^28^. The redox couple is situated at 0.289 V/0.153 V (vs saturated calomel electrode SCE) at 10 mV s^−1^ which is almost well consistent with the previous report (0.3 V/0.16 V)^12^. The slight difference in redox peak potential is mainly due to different morphology of nanostructure material^29^. This pair of peaks are mainly attributed to valence state changes related to Co^3+^/Co^4+^ as well as M^2+^/M^3+^ transitions (where M represents Ni or Co) on the surface of the electrode materials, where fast and reversible Faradaic reactions occurs^30^. The redox peaks potential of Co^3+^/Co^4+^ and M^2+^/M^3^ are so close that often in the CV pattern redox peaks observed an overlapping one^30^. Even at higher scan rate, CV curves indicate the well defined redox peaks which indicates porous NiCo_2_O_4_ microspheres are capable to sustain the fast redox reaction (Fig. 3a). CV curves also depicts that the current density increases and peak potential shifts slightly with the increase of scan rate which is also a strong indicative of low polarization and high power characteristics of electrode material. The pseudocapacitive behavior of nickel cobaltite (NiCo_2_O_4_) in alkaline electrolyte are based on following equations (2 and 3) 30.


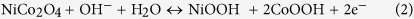



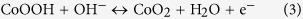


The specific capacitances of porous NiCo_2_O_4_ microspheres calculated from CV curves using equation (S2) at different scan rates (Fig. 3b). The porous NiCo_2_O_4_ microspheres exhibit specific capacitance of 774 F g^−1^ at scan rate of 2 mV s^−1^ (Fig. 3b). Figure 3b also indicates that capacitance decreases with the increase of scan rate which can be attributed to low diffusion of electrolyte ions. At higher scan rate only the outer surface can contribute to charge-discharge process which results in the reduction of utilization of electroactive materials. However in our case the porous NiCo_2_O_4_ microspheres still show high specific capacitance of 405 F g^−1^ at scan rate of 100 mV s^−1^. It can be attributed to high surface area, large pore volume and small crystallite size which can sustain the pseudocapacitive behaviour of electrode material even at higher scan rates. The porous NiCo_2_O_4_ microspheres demonstrated excellent rate capability with the capacitance retention of around 52.3% at 100 mV s^−1^ which is itself quite significant and comparable to few reported electrode materials such as Co_3_O_4_ nanoparticles (46% at 50 mV s^−1^)^31^, NiCo_2_O_4_NSs@HMRAs (50% at 50 mV s^−1^)^32^, NiO (59% at 5 mA cm^−2^)^33^, NiCo_2_O_4_ nanoparticles (37% at 100 mV s^−1^)^34^, Amorphous Mn_3_O_4_ (47% at 100 mV s^−1^)^35^, and Fe_3_O_4_-MnO_2_ (68% at 100 mV s^−1^)^36^. CV curves of active carbon at different scan rate ranging from 2 to 30 mV s^−1^ & 40 to 100 mV s^−1^ within the potential window −1.0 to 0 V vs saturated calomel electrode (Figs 3c and S9). The shapes of CV curves are quasi-rectangular indicating the electrical double layer capacitive behaviour. The area of CV curves increases with the increase of scan rate indicating the higher degree of reversibility and good rate capability. The corresponding specific capacitances of active carbon calculated using equation (S2) at different scan rates (Fig. 3d).

To fabricate asymmetric supercapacitor device, it is important to find the mass balance between the positive and negative electrode. The mass balance between them can be find out by balancing the charge storage on them using equation (4) 37.


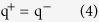


The charge storage on the electrode is proportional to the mass, specific capacitance and voltage window of the electrode material according to equation (5).


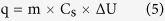


The mass balance between the positive and negative electrode can be expressed by equation (6).


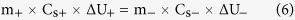


where C_s+_ & C_s−_ are the specific capacitances, ΔU_+_ & ΔU_−_ are the optimum voltage windows and m_+_ & m_−_ are the active masses for positive and negative electrodes respectively. The mass balance between the positive and negative electrodes is calculated to be 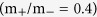.

**Figure 4:**
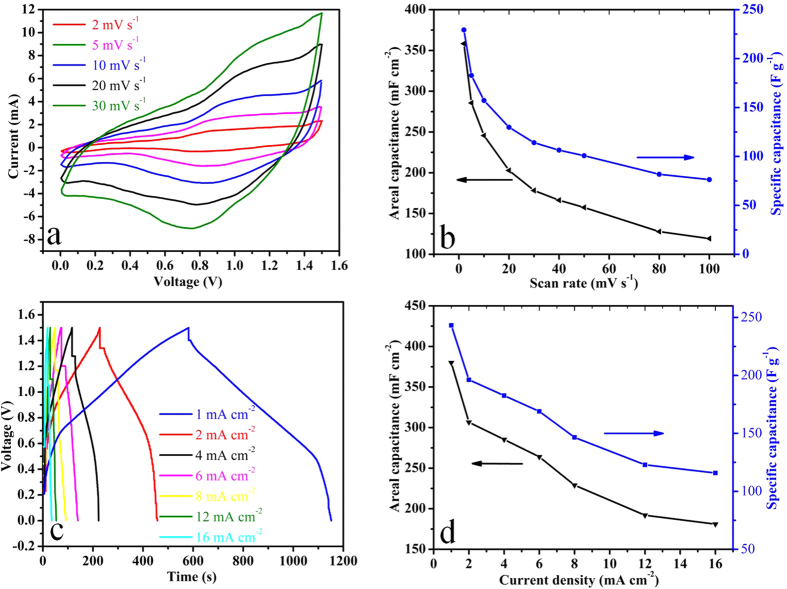
(**a**) CV curves of the asymmetric device collected at various scan rates, (**b**) areal capacitance & specific capacitance of the asymmetric device calculated as a function of scan rate based on CV curves, (**c**) CP curves of the asymmetric device collected at different current densities & (**d**) areal capacitance & specific capacitance of the asymmetric device calculated as a function of current density based on CP curves.

CV was also used to define the working potential window of ASC device. CV curves of asymmetric device at different scan rate ranging from 2 to 30 mV s^−1^ & 40 to 100 mV s^−1^ (Figs 4a and S10). CV curves present well defined reduction oxidation peaks with excellent reversibility at all scan rates within the potential window of 1.5 V which is an indication of good charge propagation within the electrodes. CV indicates the optimum potential window for the operation of asymmetric device is 0 to 1.5 V. The shape of CV curves remains unaffected even at higher scan rate which is an indication of ability of device to sustain high charge-discharge process with excellent reversibility^38^. It is also evident from CV curves with the increase of scan rate, the linear increase or decrease of peak current intensity are observed as a function of square root of scan rate (Fig. S11) which is also an indicative that the redox reaction is a diffusion controlled process. In order to determine the effect of Ni foam on the electrochemical performance of supercapacitor device. Supercapacitor device was fabricated by assembling two pieces of bare Ni foam face to face in the same way as that used for assembly of coated Ni foam. CV curves of device of bare Ni foam (Fig. S12) indicate that the substrate has no contribution towards the overall performance of fabricated device. CV was further employed to calculate the areal and specific capacitances of asymmetric device at different scan rate ranging from 2 to 100 mV s^−1^ within a potential range of 0 to 1.5 V (Fig. 4b) using equation (S3 and S4) respectively. The asymmetric device exhibit areal & specific capacitances of 358.3 mF cm^−2^ & 229.3 F g^−1^ at 2 mV s^−1^ (Fig. 4b). The capacitance decreases with the increase of scan rate because at higher scan rate diffusion of ions occurs mainly into the outer regions of the pores, while at lower scan rate, both inner and outer surfaces are accessed by the electrolyte ions which leads to high capacitance at low scan rate^39^.

The electrochemical performance of asymmetric device was also further explored using galvanostatic charge-discharge measurements. CP curves of asymmetric device at different current densities ranging from 1 to 16 mA cm^−2^ within the potential window of 0 to 1.5 V are demonstrated in Fig. 4c. CP curves shows non linear increase and decrease of potential with time which indicates the existence of pseudocapacitive behaviour of asymmetric device which is in accordance with the CV analysis. The discharge profile can be divided into two parts resistive and the capacitive. The voltage drop at the start of discharge cycle represents the resistive part which arises due to equivalent series resistance of device. CP curves of fabricated asymmetric device (Fig. 4c) are highly symmetric in comparison with the reported CP curves of few asymmetric devices which can be attributed to excellent kinetic reversibility during electrochemical reaction in porous microsphere structure^5^,^32^,^35^,^40^,^41^,^42^. Symmetrical CP curves are also a strong indicative of excellent supercapacitor behaviour of our fabricated asymmetric device. The large pore volume (0.46647 cm^3^ g^−1^) of porous microspheres acts as ion-buffering reservoirs which also plays an important role in enhancing the reversibility by reducing the anions mean free path and by facilitating the faster ionic and electronic kinetics^12^,^43^.

**Figure 5:**
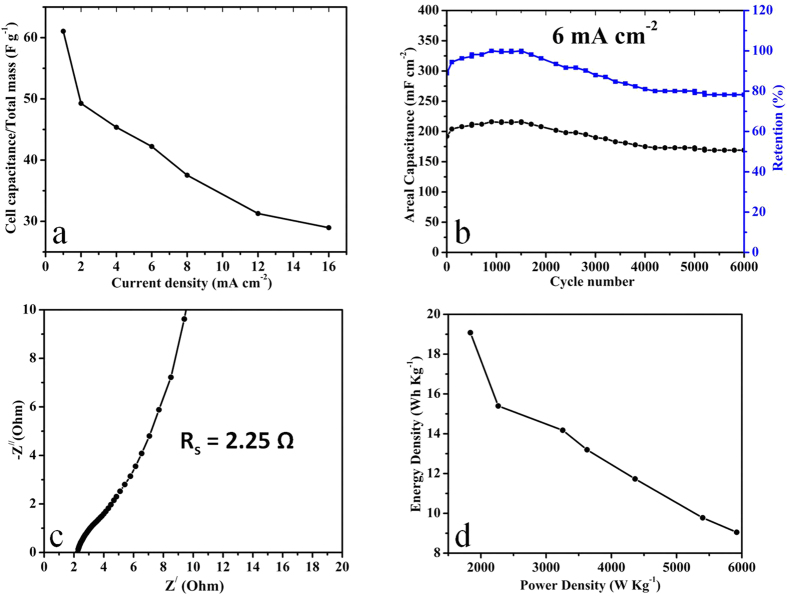
(**a**) Full cell capacitance per unit total mass of the asymmetric device at different current densities, (**b**) cycling performance test of the asymmetric device for 6000 cycles at a current density of 6 mA cm^−2^, (**c**) EIS of the asymmetric device measured at the open circuit potential in the frequency range of 100 KHz to 10 mHZ & (**d**) Ragone plot of the asymmetric device.

Figure 4d presents the areal and specific capacitances at different current densities calculated from the CP curves using equations (S5 and S6) respectively. The asymmetric device exhibit areal & specific capacitances of 380 mF cm^−2^ & 243.2 F g^−1^ at current density of 1 mA cm^−2^. It is also worthwhile to mention that the areal and specific capacitances values obtained from charge-discharge measurements are almost well consistent with that of CV results. Figure 4d depicts that the capacitance decreases with the increase of current density which can be attributed to IR drop and activation polarization due to which inner active sites are unable to participate in the electrochemical reaction at higher current densities^39^. The areal capacitance achieved (380 mF cm^−2^) in our work is the maximum value attained for asymmetric device based on pure NiCo_2_O_4_ material and it is also much higher than the few other reported asymmetric devices^33^,^44^,^45^. The maximum areal capacitance of few devices are NiO//RGO (248 mF cm^−2^ at 1 mA cm^−2^)^33^, NiO//AC (100 mF cm^−2^ at 2 mV s^−1^)^44^, Ni//AC (250 mF cm^−2^ at 2 mV s^−1^)^44^, MnO//AC (30 mF cm^−2^ at 20 mV s^−1^)^45^. The asymmetric device exhibit excellent areal capacitance retention of 48% at much higher current density 16 mA cm^−2^ (Fig. 4d) which is quite significant in comparison with the few reported asymmetric devices such as NiO//RGO (73% at 5 mA cm^−2^)^33^, NiO//AC (12% at 100 mV s^−1^)^44^, Ni//AC (32% at 100 mV s^−1^)^44^, MnO//AC (43% at 500 mV s^−1^)^45^. The full cell capacitance per unit total mass (C/M) of asymmetric device calculated by using equation (S1) is 61.0 F g^−1^ at 1 mA cm^−2^ (Fig. 5a). Despite being high total mass loading of (6.25 mg), the (C/M) is comparable or even better than few reported asymmetric devices such as hierarchical porous NiO//porous carbon (38 F g^−1^)^46^, porous Ni//AC (34 F g^−1^)^44^, NiO//RGO (50 F g^−1^)^33^, NiCo_2_O_4_ NSs@HMRAs//AC (50 F g^−1^)^32^, Co_3_O_4_//AC (54.8 F g^−1^)^29^, Co_3_O_4_@MWCNTs//AC (69 F g^−1^)^29^, AC//NaMnO_2_ (38.9 F g^−1^)^9^, AC//K_0.27_ MnO_2_·0.6H_2_O (56.22 F g^−1^)^8^, AC// Ni_3_S_2_/MWCNT-NC (55.8 F g^−1^)^40^, and PPy@MoO_3_//AC(64 F g^−1^)^7^. The asymmetric device demonstrated excellent full cell capacitance retention of around 48% which is comparable to few reported asymmetric devices such as Co_3_O_4_//AC (46%)^29^, NiCo_2_O_4_NSs@HMRAs//AC (41%)^32^, Co_3_O_4_@MWCNTs//AC (70%)^29^, AC//NaMnO_2_ (67%)^9^, AC//K_0.27_ MnO_2_·0.6H_2_O (69%)^8^, AC// Ni_3_S_2_/MWCNT-NC (62%)^40^, MoO_3_//AC (18%)^7^, and PPy@MoO_3_//AC (60%)^7^. It is also worthwhile to attain the optimum performance with high mass loading which is the critical factor regarding the practical application of device^47^. The cycling performance is the key factor which indicates the stability of electrode for a long time usage. The cyclic test was performed at 6 mA cm^−2^ to explore the endurance of asymmetric device for long time usage (Fig. 5b). Asymmetric device demonstrate excellent cycling stability with capacitance retention of 78% after 6000 cycles (Fig. 5b). The capacitance retention is comparable or even better than few reported asymmetric devices such as NiO//C (50% after 1000 cycles)^46^, Ni(OH)_2_–MnO_2_//RGO (76% after 3000 cycles)^48^, NiO//Ru_0.35_V_0.65_O_2_ (83.5% after 1500 cycles)^42^, and PPy@MoO_3_//AC (83% after 600 cycles)^7^. The cyclic test indicates that the areal capacitance increases initially in the first few hundred cycles which is attributed to the activation of electrode materials after that the asymmetric device demonstrate excellent capacitance retention^11^,^13^. CP curves for few initial, intermediate and final cycles at 6 mA cm^−2^ are presented in Fig. S13. The cyclic test also indicates its excellent applicability for long time usage. The capacity fading during cycling test may be due to the mechanical stress on the electrode material during insertion or de-insertion of electrolyte ions during charging-discharging process^43^. The reversibility of asymmetric device was further explored by measuring the Coulombic efficiency using equation (S11). The asymmetric device demonstrate excellent Coulombic efficiency of around 96% for 6000 cycles which shows its higher degree of reversibility (Fig. S14). CP curves for few cycles at current densities 1, 2, & 4 mA cm^−2^ are also presented in Fig. S15. The electrochemical impedence spectroscopy of asymmetric device was also carried out to calculate its equivalent series resistance (R_s_) and charge transfer resistance (R_ct_) (Fig. 5c). R_s_ includes cumulative resistance contributed from electroactive materials, electrolyte and interface between electrolyte/electrode^43^. The impedence behaviour (Fig. 5c) can be further subdivided into high and low frequency regions. In the high frequency region, the intercept of semicircle with the real axis represents the R_s_ and the width of semicircle plotted is an indicative of charge transfer resistance R_ct_^49^. In the low frequency region the slope of impedence plot with the real axis is more than typical Warburg angle of 45° which is an indicative of low Warburg impedence and higher capacitive behaviour due to more rapid ions or electrolyte diffusion to the electrode material. In the high frequency region (Fig. 5c) the width of semicircle is very small which indicates the charge transfer resistance caused by Faradaic reaction and electrochemical double layer capacitance is very small which is also an indicative of high performance characteristics of device. It can also be inferred from the graph the R_s_ and R_ct_ are 2.25 and 0.15 Ω respectively. Equivalent series resistance R_s_ was also responsible for the IR drop which was observed in CP measurements (Fig. 4c). The relatively low values of R_s_ & R_ct_ represent the higher diffusion and migration pathways for electrolyte ions during charge/ discharge processes which are responsible for the good electrochemical performance of asymmetric device as evidenced from both CV and CP measurements. Energy density and maximum power density of asymmetric device are calculated from CP curves at different current densities by using equations (S12 and S13) respectively and the typical Ragone plot is shown in Fig. 5d. Table S2 summarizes the detail values of energy and maximum power densities at different current densities. The asymmetric device exhibits energy density of 19.1 Wh kg^−1^ and maximum power density of 1839 W kg^−1^ at the current density of 1 mA cm^−2^. Even at higher current density of 16 mA cm^−2^, asymmetric device still delivers energy density of 9.1 W h Kg^−1^ and maximum power density of 5921 W Kg^−1^. The results indicate that the asymmetric device can be operated within the large power range with sustainable energy density. The calculated energy density is higher than or comparable to few reported asymmetric devices such as Ni-Co oxide//AC (12 W h Kg^−1^)^17^, porous NiO//porous carbon (11.875 W h Kg^−1^)^46^, Hierarchical NiCo_2_O_4_ nanosheets@ hollow microrod arrays//AC (15.42 W h kg^−1^)^32^, and a-MnO_2_// PEDOT (13.5 W h Kg^−1^)^50^, AC// Ni_3_S_2_/MWCNT-NC (19.8 W h kg^−1^)^40^, AC//NaMnO_2_ (19.5 W h Kg^−1^)^9^, AC//K_0.27_ MnO_2_·0.6H_2_O (25.3 W h Kg^−1^)^8^, and Co_3_O_4_//AC (21.0 W h Kg^−1^)^29^.

**Figure 6:**
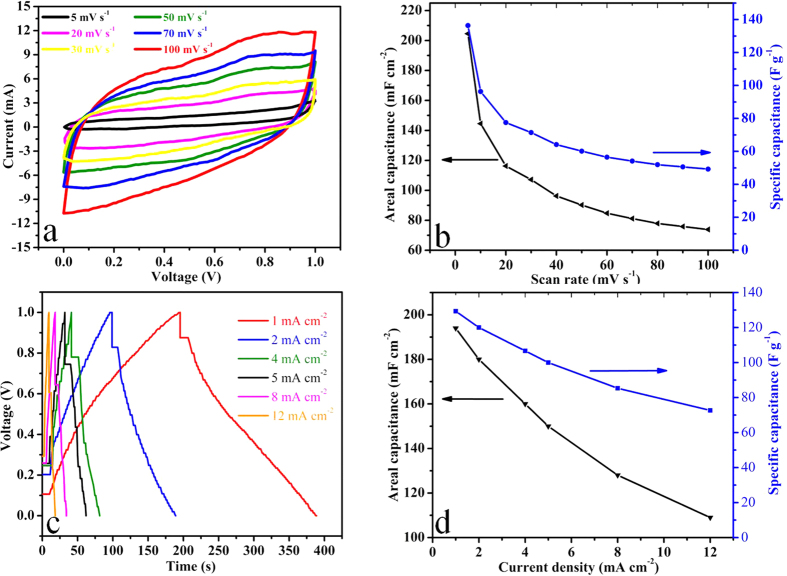
(**a**) CV curves of the symmetric device collected at various scan rates, (**b**) areal capacitance & specific capacitance of the symmetric device calculated as a function of scan rate based on CV curves, (**c**) CP curves of the symmetric device collected at different current densities & (**d**) areal capacitance & specific capacitance of the symmetric device calculated as a function of current density based on CP curves.

**Figure 7:**
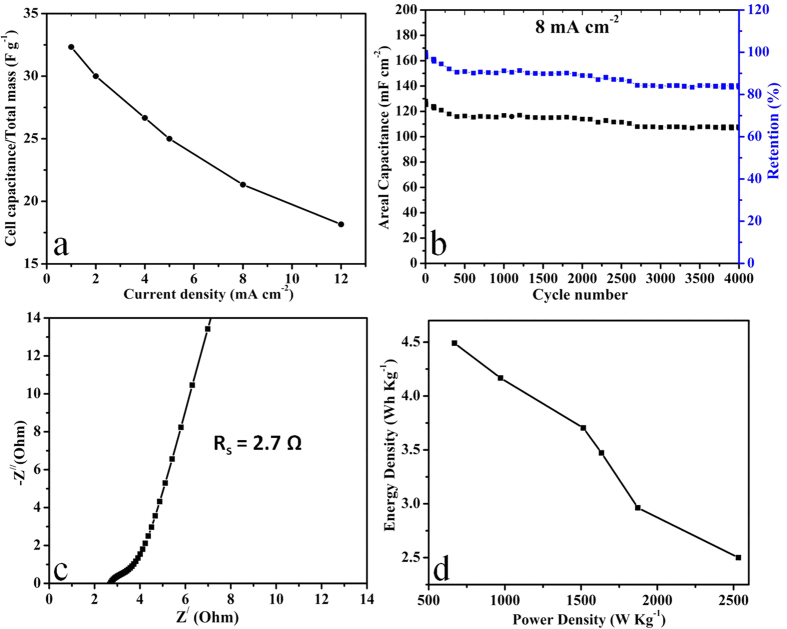
(**a**) Full cell capacitance per unit total mass of the symmetric device at different current densities, (**b**) cycling performance test of the symmetric device for 4000 cycles at a current density of 8 mA cm^−2^, (**c**) EIS of the symmetric device measured at the open circuit potential in the frequency range of 100 KHz to 10 mHZ & (**d**) Ragone plot of the symmetric device.

For comparison we also fabricated the symmetric device of porous NiCo_2_O_4_ microspheres in the same way as that used for asymmetric device. The main objective is to explore the potential of porous NiCo_2_O_4_ as an electrode material of symmetric device. In the future perspective it is also important to harness its working principle and operational limitation. Figure 6a presents CV curves of symmetric device at different scan rate ranging from 5 to 100 mV s^−1^. The shape of CV curves remains unchanged with no distortion depicts that the capacitive behaviour of symmetric device is mainly governed by the non-Faradaic electrical double layer capacitance^51^. The area of CV curves increases with the increase of scan rate but its shape remains unchanged even at higher scan rate which is an indication of kinetic reversibility of symmetric device. The absence of redox peaks in CV pattern of symmetric device indicates the symmetric device is charged and discharged at pseudoconstant rate^52^,^53^. Although CV scans of as-synthesized material in three electrode cell configuration has already indicated the well defined pseudocapacitive behaviour (Fig. 3a). We can conclude that two electrode symmetric devices are primarily non-Faradaic in nature because the dominant behaviour of capacitance is governed by electrical double layer capacitance rather than the pseduocapacitance^51^,^54^. The areal and specific capacitances of symmetric device are also calculated from CV curves at different scan rate by using equation (S7 & S8) respectively, the results are plotted in Fig. 6b. The symmetric device exhibit the areal and specific capacitances of 204.55 mF cm^−2^ and 136.3 F g^−1^ at scan rate of 5 mVs^−1^ respectively. CP curves of symmetric device at different current densities ranging from 1 to 12 mA cm^−2^ within the potential window of 0 to 1.0 V are demonstrated in Fig. 6c. CP curves exhibits linear increase and decrease of potential with time which indicates the existence of double layer capacitive behaviour of symmetric device which is also in accordance with its CV analysis (Fig. 6a). Figure 6d presents the areal and specific capacitances at different current densities calculated from the CP curves using equation (S9 & S10) respectively. The symmetric device exhibit areal & specific capacitances of 194 mF cm^−2^ & 129.33 F g^−1^ at current density of 1 mA cm^−2^. The full cell capacitance per unit total mass (C/M) of symmetric device calculated by using equation (S1) is 32 F g^−1^ at 1 mA cm^−2^ (Fig. 7a). Secondly our results are much better than many previous reported symmetric supercapacitor devices^45^,^55^,^56^,^57^,^58^. The more specifically MnO (28.3 mF cm^−2^ at 20 mV s^−1^)^45^, active carbon AC(15.6 mF cm^−2^ at 20 mV s^−1^)^45^, ZnCo_2_O_4_ nanorods (36 mF cm^−2^ at 1 mA cm^−2^)^55^, NiCo_2_O_4_ nanowires (160 mF cm^−2^ at 1 mA cm^−2^)^56^, hydrogenated ZnO core-shell nanocables (26 mF cm^−2^ at 0.25 mA cm^−2^)^57^, MnO_2_-polypyrrole hybrid nanofilm (25.9 mF cm^−2^ at 0.2 mA cm^−2^)^58^. The symmetric device capacitance retention is around 56% at 12 mA cm^−2^ (Fig. 7a) which is much higher than few reported symmetric devices such as MnO (19.9% at 200 mV s^−1^)^45^, active carbon AC (56.0% at 200 mV s^−1^)^45^, ZnCo_2_O_4_ nanorods (25.0% at 2.0 mA cm^−2^)^55^, hydrogenated ZnO core-shell nanocables (48.0% at 5.0 mA cm^−2^)^57^, and MnO_2_-polypyrrole hybrid nanofilm (50.08% at 250 mV s^−1^)^58^. The higher areal capacitance and its excellent retention indicate the superior device performance in comparison with the few reported symmetric devices^45^,^55^,^56^,^57^,^58^.

The cyclic test was performed at 8 mA cm^−2^ to explore its cycling stability for long time usage which indicates excellent capacitance retention of 80% after 4000 cycles (Fig. 7b). CP curves of few initial, intermediate and final cycles at 8 mA cm^−2^ are presented in Fig. S16. The symmetric device demonstrate excellent Coulombic efficiency of around 100% for 4000 cycles which shows its higher degree of reversibility (Fig. S17). CP curves for few cycles at current densities 1, 2, 4 & 5 mA cm^−2^ are also presented in Fig. S18. Electrochemical impedence spectroscopy of symmetric device was also performed to quantify the R_s_ and R_ct_ (Fig. 7c).The impedence behaviour of symmetric device is almost similar to that of asymmetric device (Fig. 5c) with only small increase in charge transfer resistance R_ct_ (0.61 Ω) and equivalent series resistance R_s_ (2.70 Ω) which also indicates the higher conductivity and low ion diffusion resistance of asymmetric cell in comparison with the symmetric cell. Energy density and maximum power density of symmetric device are calculated from CP curves at different current densities by using equations (S12 and S13) respectively and the typical Ragone plot is shown in Fig. 7d. The symmetric device exhibit energy density of 4.5 Wh kg^−1^ and maximum power density of 669.0 W kg^−1^ at the current density of 1 mA cm^−2^ (Fig. 7d). Even at higher current density of 12 mA cm^−2^, symmetric device still delivers energy density of 2.5 W h Kg^−1^ and power density of 2532.5 W Kg^−1^ (Fig. 7d). Table S3 summarizes the detail values energy and maximum power densities at different current densities. The calculated energy density is higher than or comparable to few reported symmetric supercapacitor devices such as graphene//graphene (2.8 Wh kg^−1^)^59^, MnO_2_ nanowire/graphene composite (MGC//MGC) (5.2 Wh kg^−1^)^59^, active carbon AC//AC (3.6 Wh kg^−1^)^6^, Au-MnO_2_/CNT // Au-MnO_2_/CNT (4.5 Wh kg^−1^)^60^, MnO_2_/CNT// MnO_2_/CNT (2.9 Wh kg^−1^)^60^, and MnO_2_//MnO_2_ (1.9 Wh kg^−1^)^6^. The higher energy density than active carbon and graphene based symmetric devices also indicates that it can be implemented as the negative electrode material for asymmetric devices.

## Discussion

In this work, porous NiCo_2_O_4_ microspheres are synthesized by using facile, ultrafast, cost effective and environmental friendly microwave heating method. This method also offers some interesting features such as economy of reagents, reproducibility and moderate synthesis condition. This process can easily be scaled up for the industrial scale production of high quality product. High performance asymmetric (NiCo_2_O_4_ //AC) & symmetric (NiCo_2_O_4_// NiCo_2_O_4_) supercapacitor devices are successfully fabricated inside the coin cell CR2032 with high mass loading of active materials using 2M KOH aqueous electrolyte. ASC device operates within a potential window of 1.5 V and exhibits high areal capacitance of 380 mF cm^−2^ (full cell capacitance per unit total mass of 61 F g^−1^) and energy density of 19.1 Wh kg^−1^ at current density of 1 mA cm^−2^. Whereas the SSC device operates within a potential window of 1.0 V and exhibits high areal capacitance of 194 mF cm^−2^ (full cell capacitance per unit total mass of 32 F g^−1^) and energy density of 4.5 Wh kg^−1^ at current density of 1 mA cm^−2^. The development of supercapacitor devices with high areal capacitance is quite significant considering its great importance for applications like consumer electronics, chip & IC packaging and MEMS module. The achieved values of areal capacitances for both devices are the highest for pure NiCo_2_O_4_ material. The enhancement of areal capacitance can be attributed to porous NiCo_2_O_4_ microspheres structure which have relatively high specific surface area (119.68 m^2^ g^−1^), large pore volume (0.46647 cm^3^ g^−1^), mesoporous porosity, narrow pore size distribution (4.85–15 nm) and small crystallite size (8 nm). The high specific surface area and small crystallite size provide the maximum utilization of electroactive material during electrochemical reaction despite being high mass loading. CV & CP measurements indicate that the existence of pseudocapacitive behaviour in the asymmetric devices and double layer capacitive behaviour in the symmetric devices. CP curves of both devices (Figs 4c and 6c) are highly symmetric which indicates their excellent supercapacitor behaviour. It means that area of charge part is almost the same as that of discharge part (Figs 4c and 6c), which indicates the excellent efficiency of fabricated devices. The energy density associated with asymmetric device is 4.25 times higher than the symmetric device working in the same aqueous electrolyte. The main reasons of higher energy density of asymmetric device are due to its wide operating voltage window and higher full cell capacitance per unit total mass (C/M) than that of its symmetric device. The cyclic performance of ASC & SSC devices indicates excellent capacitance retention of 78 & 83% after 6000 & 4000 cycles at current densities of 6 & 8 mA cm^−2^ respectively. The excellent cyclic stability can be attributed to porous nanostructures which minimizes the strain generated during ion insertion/desertion process. Table S2 also indicates that the ASC device can be operated within the large power range with sustainable energy density. Both asymmetric & symmetric devices provide an excellent options as an energy storage devices with high power density and sustainable energy density for specific applications. The successful fabrication of symmetric supercapacitor also indicates the potentialities of porous NiCo_2_O_4_ microspheres that can be used as the negative electrode material for the futuristic energy storage asymmetric devices. We can conclude that the aqueous based asymmetric device (NiCo_2_O_4_ //AC) is the rational option to enhance the areal capacitance & energy density by increasing its voltage window up to 1.5 V. In summary our work also open up the opportunity to develop the energy storage devices with high areal capacitance to meet up the demand of future miniaturized electronics.

## Methods

### Materials synthesis

All chemicals were of analytical grade and were used directly after purchase without further purification. In a typical synthesis, 2 mmol of Ni(NO_3_)_2_·6H_2_O, 4 mmol of Co(NO_3_)_2_·6H_2_O and 120 mmole of urea were dissolved into a 100 ml of de-ionized water with constant magnetic stirring at room temperature for 60 minutes to form a clear pink solution. The solution was then transferred to a 300 ml 3 necked flask then placed in the microwave heating oven (MAS II, SINEO), and heated at 100 °C under medium high mode power of 300 watt for 30 minutes. After reaction the solution was allowed to cool down naturally and standing for 24 hrs. The solution was centrifuged several times at 12000 rpm for 4 minutes using de-ionized water and absolute ethanol to collect the precipitates. The precipitates were then dried at 100 °C in air environment overnight. The as-prepared precursor was calcinated at 300 °C in atmospheric environment for 210 minutes to transform it into porous NiCo_2_O_4_ microspheres.

### Structural characterization

Thermogravimetric analysis (TGA) was carried out to determine the calcination temperature of as-synthesized precursor using TGA-50 (Shimadzu) in a flowing nitrogen environment with a heating rate of 10 °C min^−1^. The phase analysis of samples were carried out using X-ray diffraction (XRD; Philips X’Pert Pro MPD). XRD data were recorded using Ni filtered Cu Kα radiation (λ = 1.54178 Å) source. The accelerating voltage and current of XRD unit were set at 40 KV and 40 mA respectively and scans were performed in a 2θ range from 10° to 80° at a scanning speed of 0.02°s^−1^. The morphology and microstructure of the samples were analysed using field-emission scanning electron microscope (FESM, Hitachi S4800) and transmission electron microscopy (TEM, Tecnai G2 F20, U-TWIN). The compositional analysis of the sample was carried out using energy dispersive X-ray spectroscopy (EDX) attached with (FESEM, Hitachi S4800). Fourier transform infrared spectra (FTIR) were recorded on a Thermo Scientific Nicolet 6700 FT-IR spectrometer in the range of 500–4000 cm^−1^. The valence states of different elements of calcinated product were examined by X-ray photoelectron spectroscopy (XPS) using Thermo Scientific ESCALAB-250Xi spectrometer with Al K_α_ (1,486.6 eV) radiation source. XPS spectra were recorded at the total instrumental resolution of 1.18 eV for Al K_α_ excitation source. The specific surface area was calculated using the Brunauer–Emmett–Teller (BET) method, while the pore size distribution, average pore diameter and pore volume were calculated from the desorption branches based on the Barrett–Joyner–Halenda equation using Micromeritics ASAP 2020 M analyzer.

### Fabrication & electrochemical characterization of asymmetric supercapacitor

The working electrodes were prepared on well cleaned nickel foam (11 mm diameter) using traditional slurry coating method. Nickel foam was cleaned by ultrasonication sequentially in acetone, 1 M HCl solution, deionized water and ethanol for 30 minutes each and then dried at 100 °C for 24 hrs for subsequent use. The positive electrode was prepared by mixing 80 wt % of as-prepared porous NiCo_2_O_4_ microspheres, 14 wt % acetylene black and 6 wt % polytetrafluoroethylene (PTFE) using mortal pestle and then a few drops of ethanol was added to above mixture to make its slurry. The slurry was pasted on to Ni foam (11 mm diameter) using a microinjector. The working electrodes were dried at 80 °C for 24 hrs in a vacuum oven and then pressed at 10 MPa for 30 seconds and further dried overnight. The negative electrode of active carbon was prepared in the same way as that used for positive electrode except by using 80 wt % active carbon instead of porous NiCo_2_O_4_ microspheres. To determine the mass balance between positive and negative electrode, cyclic voltammetry (CV) measurements of porous NiCo_2_O_4_ and active carbon were carried out initially in a standard three-electrode cell which consists of Hg/HgCl (Saturated KCl solution) as the reference electrode, Pt wire as the counter electrode, and porous NiCo_2_O_4_/Ni or active carbon/Ni foam as the working electrode in 2.0 M KOH electrolyte. The mass ratio of positive electrode to negative electrode was around 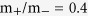. All the asymmetric devices were fabricated according to the calculated mass balance between the positive and negative electrodes. The total mass loading of positive and negative electrode was maintained around 6.25 mg. Asymmetric supercapacitor device was fabricated by assembling porous NiCo_2_O_4_/Ni foam as the positive electrode and active carbon/Ni foam as the negative electrode face to face with polypropylene membrane as separator into coin cell CR2032 in 2.0 M KOH electrolyte. While the symmetric supercapacitor device was fabricated with the same two electrodes of porous NiCo_2_O_4_/Ni foam having equal masses in the same way as that of asymmetric device. For symmetric device total mass loading of positive and negative electrode was maintained around 6.0 mg. The prior to fabrication of supercapacitor device, electrodes were immersed in 2.0 M KOH electrolyte overnight for electrochemical activation of active material. Cyclic voltammetry (CV), electrochemical impedance spectra (EIS) measurements were carried out using IM6e electrochemical workstation and galvanostatic charging–discharging or chronopotentiomerty (CP) measurements were performed via LAND CT2001 tester to investigate the electrochemical performance of fabricated devices. The electrochemical properties of both devices were investigated by CV, CP and EIS measurements in 2 M KOH electrolyte at room temperature. EIS analysis was carried out from 100 KHz to 10 mHz in a frequency sweep against the open-circuit voltage with a sinus amplitude of 5 mV. The full cell capacitance, energy density and maximum power density were calculated on the basis of total mass loading of active materials on both electrodes. The calculation method used to calculate the areal capacitance C_a_ (F cm^−2^), specific capacitance C_sp_ (F g^−1^), full cell capacitance C per unit total mass (F g^−1^), energy density E (Wh kg^−1^), maximum power density P(W kg^−1^) and Coulombic efficiency 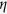 (%) are described in detail in the supporting information.

## Additional Information

**How to cite this article**: Khalid, S. *et al*. Microwave Assisted Synthesis of Porous NiCo_2_O_4_ Microspheres: Application as High Performance Asymmetric and Symmetric Supercapacitors with Large Areal Capacitance. *Sci. Rep.* **6**, 22699; doi: 10.1038/srep22699 (2016).

## Supplementary Material

Supplementary Information
