# Supplementary material for: Microwave Assisted Synthesis of Porous NiCo2O4 Microspheres: Application as High Performance Asymmetric and Symmetric Supercapacitors with Large Areal Capacitance
Source: Sci Rep. 2016 Mar 3;6:22699. doi: 10.1038/srep22699 (PMC4776212; doi:10.1038/srep22699)
Supplement: Supplementary Information [file srep22699-s1.pdf]

Supporting Information

SUBJECT AREAS: MICROSPHERES • NICKEL COBALTITE • MICROWAVE SYNTHESIS • ASYMMETRIC & SYMMETRIC SUPERCAPACITOR DEVICES • ELECTROCHEMISTRY

Correspondence and requests for materials should be addressed to C. Cao (cbcao@bit.edu.cn)

Microwave Assisted Synthesis of Porous $NiCo_2O_4$ Microspheres: Application as High Performance Asymmetric and Symmetric Supercapacitors with Large Areal Capacitance

Syed Khalid, Chuanbao Cao,* Lin Wang, Youqi Zhu

Research Center of Materials Science, Beijing Institute of Technology, Beijing 100081, P. R. China

Calculation of areal capacitance, specific capacitance, full cell capacitance, Coulombic efficiency, energy density and power density

It is important to understand the relation between specific capacitance and cell capacitance. Because in few reported published papers they have used directly the specific capacitance C_s instead of using full cell capacitance C, which gives high values of energy densities. So for better understanding we have elaborated the whole calculation procedure in detail. The specific capacitance is the capacitance per unit mass of one electrode as shown in equation (S1).[1,2]

$$C_S(F\ g^{-1}) = 4 \times \frac{C}{M} \quad \text{(S1)}$$

Where C is the measured **full cell capacitance** of two electrode cell and **M** is the **total mass** of both positive and negative electrode. The multiplier **4** adjust the capacitance of cell and the combined mass of two electrode to the capacitance and mass of one electrode.[1,2]

1. Three electrode configuration: The specific capacitance was calculated from CV curves using equation (S2) .[3]

$$C_S(F\ g^{-1}) = \frac{S}{2\Delta U \times f \times m} \quad \text{(S2)}$$

where S, f(mV s^{-1}), ΔU(V) and m(g^{-1}) are the area under the curve of CV loop, scan rate, potential window and mass of active material in the working electrode respectively.

2. Two electrode asymmetric cell configuration

The areal and specific capacitances were calculated from CV and CP curves using equation(S3 & S4) and (S5 & S6) respectively.[3-5] The mass loading of positive & negative electrode are maintained at ($m_+ = 1.788$ mg) & ($m_- = 4.47$ mg) respectively.

$$C_a(F\ cm^{-2}) = \frac{S}{2\Delta U \times f \times A} \qquad \text{(S3)}$$

$$C_s(F\ g^{-1}) = 4 \times \frac{S}{2\Delta U \times f \times A \times M} \qquad \text{(S4)}$$

where S, f(mV s^{-1}), ΔU(V), A(cm^2) and M(g^{-1}) are the area under the curve of CV loop, scan rate, potential window, area of electrode, total mass loading of both electrodes respectively.

$$C_a(F\ cm^{-2}) = \frac{I \times \Delta T_d}{A \times \Delta U} \qquad \text{(S5)}$$

$$C_s(F\ g^{-1}) = 4 \times \frac{I \times \Delta T_d}{A \times \Delta U \times M} \qquad \text{(S6)}$$

where I(mA), ΔT_d(s), A(cm^2), ΔU(V) and M(g^{-1}) are the constant discharge current, discharge time, area of electrode, discharging voltage after IR drop, mass loading of positive and negative electrode respectively.

2. Two electrode symmetric cell configuration

The areal and specific capacitances were calculated from CV and CP curves using equation (S 7 & S8) and (S9 & S10) respectively.[3,6]

$$C_a(F\ cm^{-2}) = \frac{S}{2\Delta U \times f \times A} \qquad \text{(S7)}$$

$$C_s(F\ g^{-1}) = 2 \times \frac{S}{2\Delta U \times f \times m} \qquad \text{(S8)}$$

where S, f(mV s^{-1}), ΔU(V), A(cm^2) and m(g^{-1}) are the area under the curve of CV loop, scan rate, potential window, area of electrode and **mass** of active material on **one electrode** respectively. The mass loading on one electrode (m = 3.0 mg)

$$C_a(F\ cm^{-2}) = \frac{I \times \Delta T_d}{A \times \Delta U} \qquad \text{(S9)}$$

$$C_s(F\ g^{-1}) = 2 \times \frac{I \times \Delta T_d}{m \times \Delta U} \tag{S10}$$

where I(mA), $\Delta T_d(s)$, A(cm^2), $\Delta U(V)$ and m(g^{-1}) are the constant discharge current, discharge time after IR drop, area of electrode, discharging voltage after IR drop and **mass** of active material on **one electrode** respectively. The multiplying factor of **2** was used in calculating the specific capacitance because the series capacitance was formed in two electrode symmetric devices.[7,8]

2. Calculation of Coulombic efficiency, energy density and maximum power density

Coulombic efficiency, energy density ($WhKg^{-1}$) and maximum power density (Wkg^{-1}) of both devices were calculated from the CP curves according to equation (S11, S12 & S13) respectively.[2,5,8]

$$\eta = \frac{\Delta T_d}{\Delta T_c} \times 100 \tag{S11}$$

where $\Delta T_d(s)$ and $\Delta T_c(s)$ discharge time after IR drop and charge time respectively.

$$E(WhKg^{-1}) = \frac{1}{2 \times 3.6} \times \frac{C}{M} \times (\Delta U)^2$$

By using equation (S1), the full cell capacitance per unit total mass is given by

$$\frac{C}{M} = \frac{C_s(F\ g^{-1})}{4}$$

$$E(WhKg^{-1}) = \frac{1}{2 \times 3.6} \times \frac{C_s}{4} \times (\Delta U)^2 \tag{S12}$$

$$P_{max}(Wkg^{-1}) = \frac{U^2}{4 \times M \times R_{ir}} \tag{S13}$$

where $R_{ir} = \frac{\Delta U_{ir}}{2 \times I}$

where U (V) is the maximum voltage attained during charge, R_{ir} is the internal resistance which is determined from the voltage drop at the beginning of each discharge, while the ΔU_{ir} represents the voltage drop during discharge cycle which corresponds to IR drop, I(mA) is the constant discharge current and M(Kg) is the total mass of both positive and negative electrode.

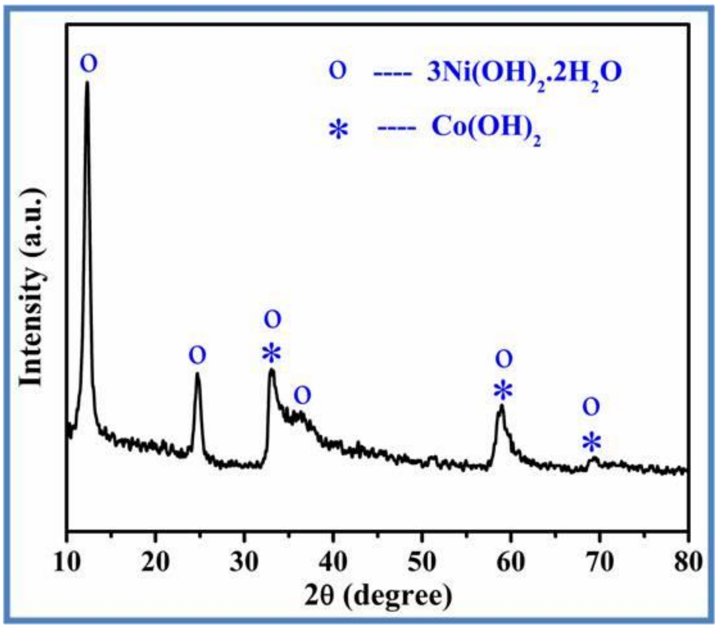

Figure S1. XRD pattern of as-synthesized precursor

All observed peaks can be well indexed to $Co(OH)_2$ and $3Ni(OH)_2.2H_2O$ according to JCPDS card no.02-0925 and 022-0444 respectively.

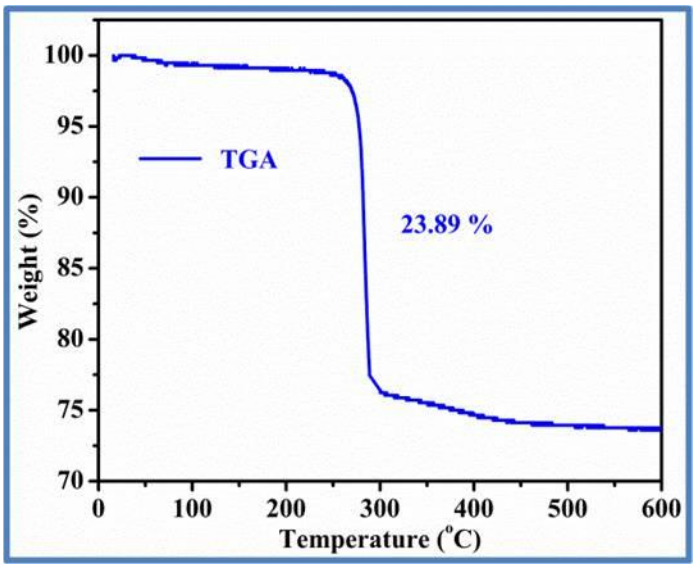

Figure S2.TGA curve of as-prepared precursor

TGA of as-prepared precursor was carried out from room temperature to 600°C in a flowing nitrogen environment as shown in Figure S2. It is evident from Figure S2 that the precursor undergoes multisep weight loss in the temperature range from RT to 300°C. In the first step, weight loss (1.25%) below 200°C can be associated with the removal of adsorbed water and intercalated water molecule.[9] The major weight loss (22.64%) which starts at 245°C and finishes at 300°C can be ascribed to the decomposition and dehydroxylation of hydroxide of nickel and cobalt to form a new stable phase.[10] At temperature greater than 300°C, there is no significant weight loss which is an indication of formation of thermally stable phase.

Table S1. The average crystallite size calculated from XRD data of porous $NiCo_2O_4$ microspheres

**Peak angle (2Θ) (°)**	**Orientation**	**FWHM (β) (°)**	**Crystallite size (nm)**
31.86	(220)	0.9618	8.6
36.552	(311)	0.958	8.7
44.481	(400)	1.035	8.3
55.442	(422)	1.1707	7.7
58.978	(511)	1.328	6.9
64.923	(440)	1.18	8.0
76.870	(533)	1.617	6.3

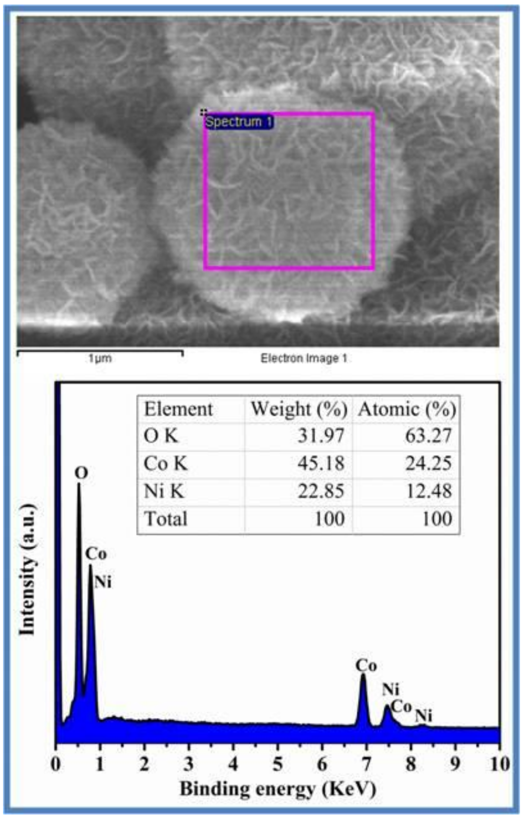

Figure S3. EDX pattern of the porous $NiCo_2O_4$ microspheres

Figure S3 presents the energy dispersive X-ray spectroscopy (EDX) spectrum of calcinated powder which shows that the molar ratio of Ni to Co is almost 1:2, which further proves the formation of pure phase of $NiCo_2O_4$.

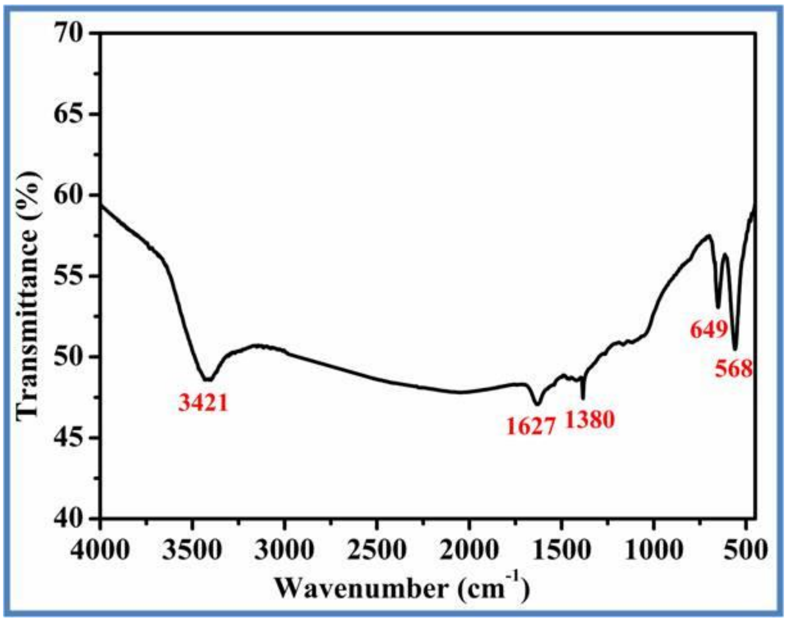

Figure S4. FTIR spectrum of porous $NiCo_2O_4$ microspheres

FTIR analysis was carried out to spot out the presence of any hydroxyl group in the calcinated powder as shown in Figure S4. FTIR analysis indicates the five characteristic bands at 3424, 1627, 1380, 649 and 568 cm^{-1}, which are well consistent with the previous reports of $NiCo_2O_4$.[11,12]

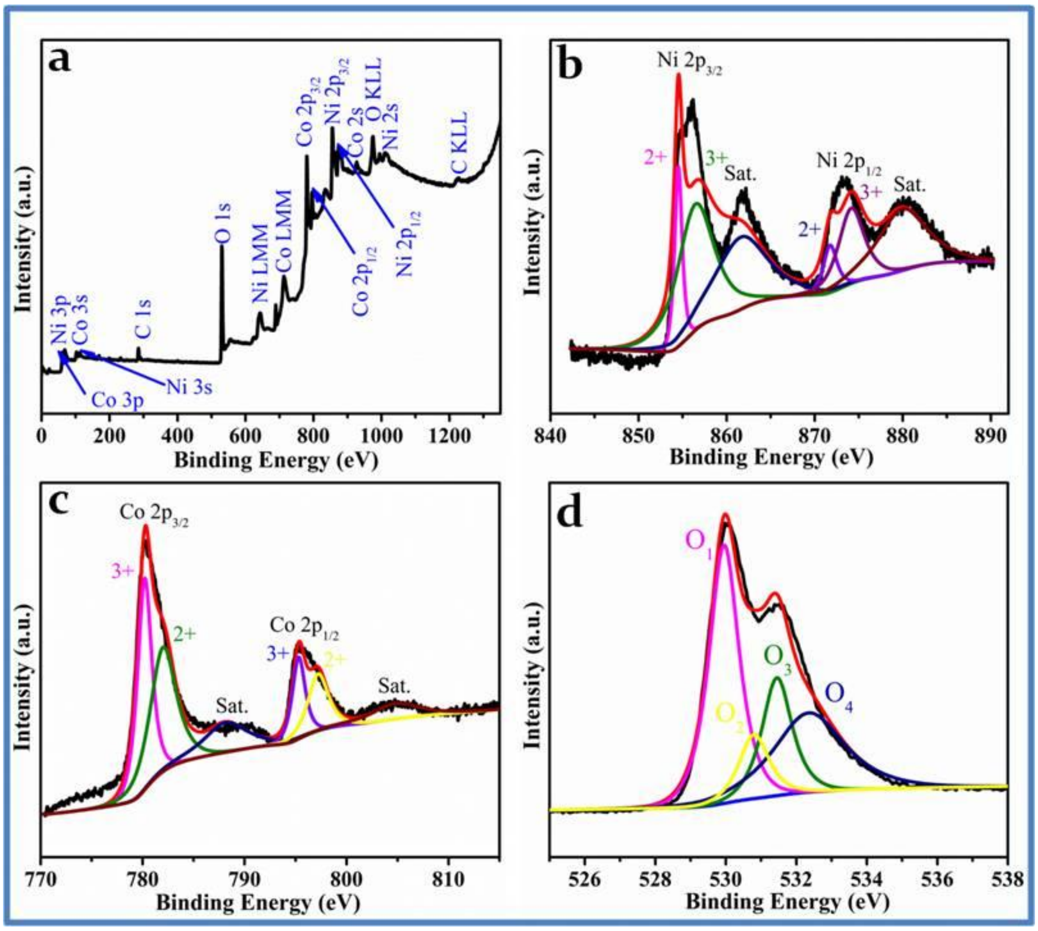

Figure S5. XPS spectra of as-synthesized porous $NiCo_2O_4$ microspheres (a) survey scan; (b) Ni 2p; (c) Co 2p; and (d) O 1s.

X-ray photoelectron spectroscopy (XPS) analysis was carried out to determine the composition and the surface electronic state of the as-synthesized porous $NiCo_2O_4$ microspheres as shown in Figure S5. It is evident from the survey spectrum that porous $NiCo_2O_4$ microspheres mainly consists of nickel, cobalt, oxygen, and carbon (as reference) species as shown in Figure S5 (a), with no any other species are detected, which is in accordance with EDS analysis. The Ni 2p spectrum is best fitted using Gaussian's method

which shows the presence of two spin-orbit doublets characteristic of Ni^{2+} and Ni^{3+},and two shakeup satellites (marked as satellite) as shown in Figure S5 (b). The fitting peaks at binding energies of 854.52 & 871.75 eV and 856.42 & 874.15 eV are ascribed to Ni^{2+} and Ni^{3+} oxidation states of nickel respectively.[13,14] Similarly the Co 2p spectrum the fitting peaks at binding energies at 782.0 & 797.26 eV and 780.2 & 795.30 are assigned to Co^{2+} and Co^{3+} oxidation states of cobalt respectively as shown in Figure S5(c).[14-16] Figure S5 (d) depicts the XPS spectrum of O 1s which can be fitted with four peaks O_1, O_2, O_3 and O_4 at binding energies of 529.0, 529.7, 531.0 and 532.5 eV respectively.

The O_1 is associated with metal-oxygen bonds, O_2 presents the oxygen in hydroxyl group, O_3 demonstrates the high number of defect sites with low oxygen co-ordination in the material with small particle size and O_4 reveals the presence of multiplicity of physi/chemisorbed water at and within the surface.[13,16-18]

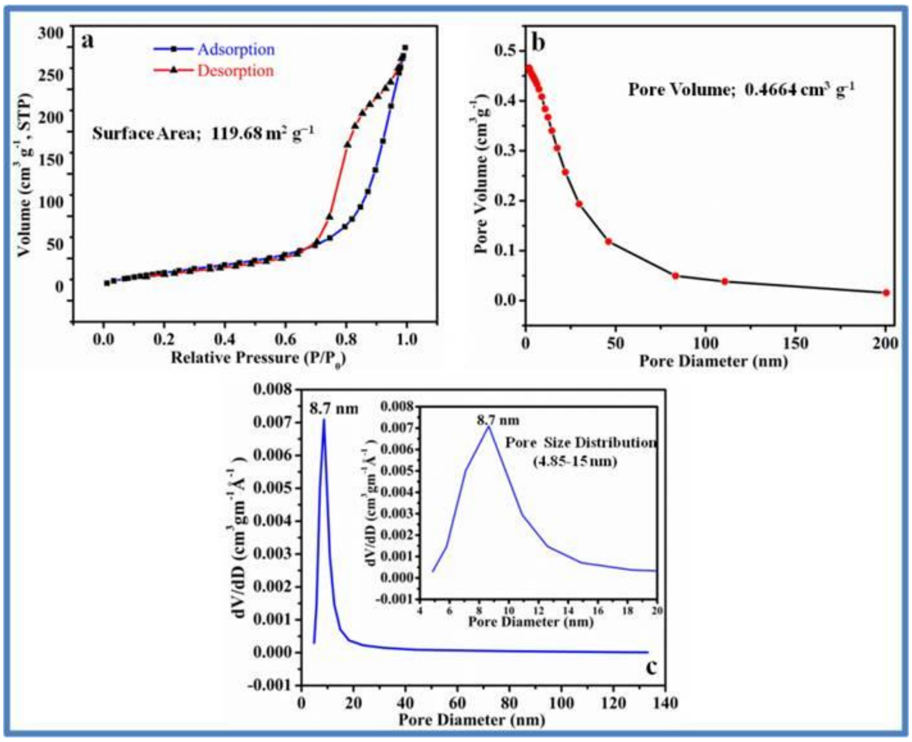

Figure S6. BET plots of porous $NiCo_2O_4$ microspheres: (a) nitrogen adsorption /desorption isotherms measured at 77 K (b) pore volume distribution, and (c) BJH pore size distribution (Inset: the enlarged view of pore size distribution)

In Figure S6 (a), the isotherm belongs to Langmuir type IV characteristics and exhibits an

obvious hysteresis loop in the range 0.7-1.0 P/P0 which indicates the presence of mesoporous structure.[19,20] The specific surface area calculated from N_2 adsorption-desorption isotherm using Brunauer-Emmett-Teller (BET) method is 119.68 $m^2\ g^{-1}$. The high specific surface area will increase the contact area at electrolyte/electrode interface which will provide abundant active sites for Faradaic reaction during electrochemical reaction. The pore volume of as-synthesized material is 0.46647 $cm^3\ g^{-1}$ as shown in Figure S6 (b). The large pore volume can serve as a reservoir for ions and also greatly enhance the diffusion kinetics within the electrode material. Figure S6 (c) depicts the pore size distribution calculated by Barrett–Joyner–Halenda model which further proves the presence of mesoporous structure with narrow and ordered distribution of pores at 8.7 nm and pore size distribution mainly centres in the range of 4.85-15 nm as shown in the inset of Figure S6 (c). The narrow and ordered distribution of pores which centres at 8.7 nm corresponds to optimum pore size for excellent electrochemical application.[21,22] The mesoporous porosity as indicated by the pore size distribution will provide the fast diffusion of ions and electron to the electrode material. Thus as-prepared porous microspheres having high specific surface area, large pore volume and narrow pore distribution could be the excellent electrode material for energy storage device.

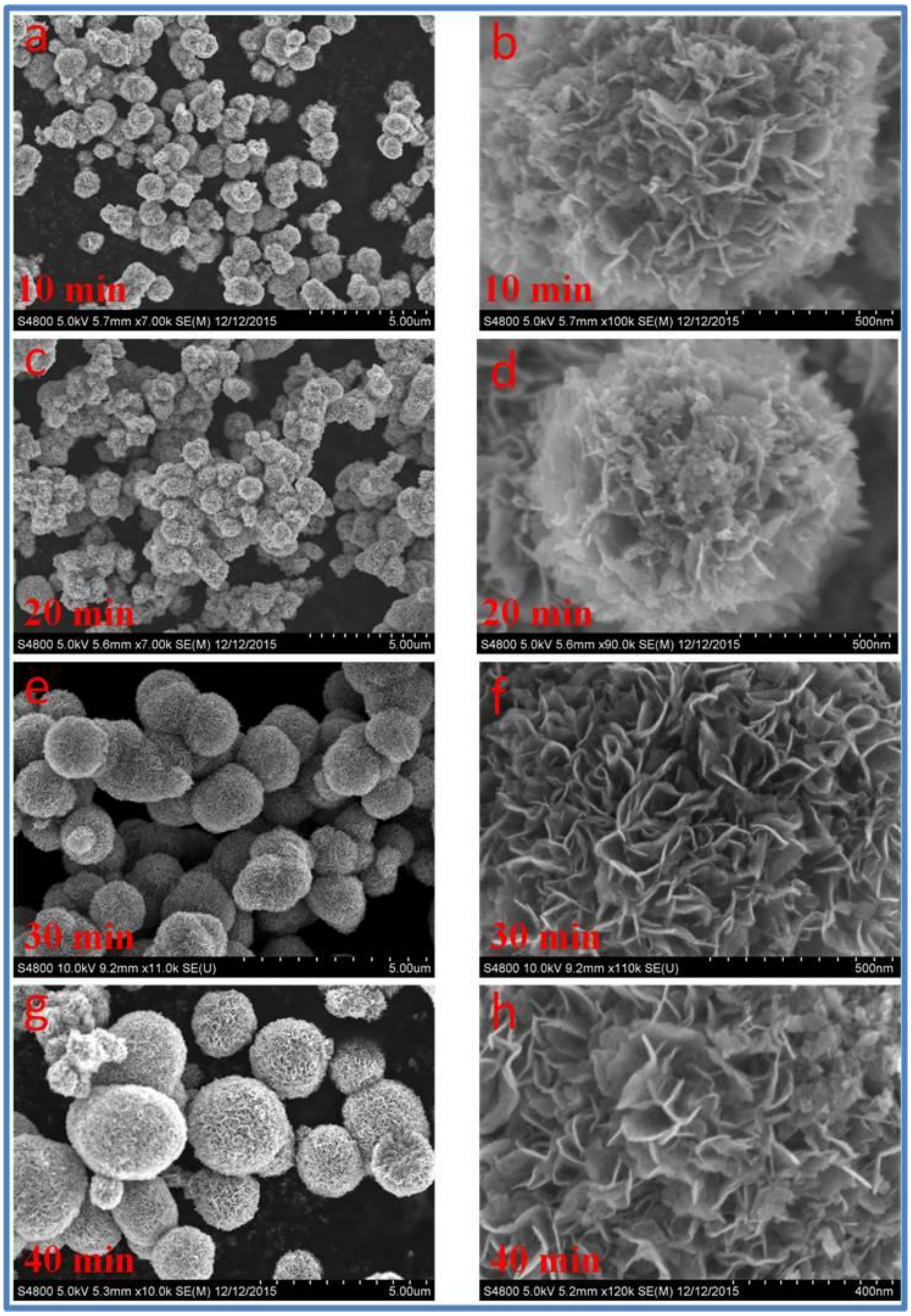

Figure S7. SEM images of porous $NiCo_2O_4$ microspheres at different synthesis time : (a & b) 10 min., (c & d) 20 min., (e & f) 30 min., and (g & h) 40 min.

SEM was used to harness the effect of microwave on the growth mechanism of microspheres in detail by employing the time dependent synthesis (10 to 40 minutes) (Figure S7). The main objective was to optimize the synthesis time which delivers high product yield with fully developed surface morphology and more uniform size distribution of microspheres. We have found that with the increase of synthesize time the product yield and average size of microspheres also enhanced. It is also evident from Figure S7 that the shape of microspheres

become well developed with enhanced number density of nanosheets with the increase of synthesize time. But SEM images also revealed that the shape of microspheres are not fully developed and not uniform by the end of synthesis duration of 10 & 20 minutes (Figure S7(a,b) & S7(c,d)). The fully developed microspheres which constituted with enhanced number density of nanosheets are formed at the synthesize time of 30 minutes (Figure S7 (e & f)). But the most importantly although the yield of product further enhanced at the synthesize duration of 40 minutes but the size distribution of microspheres are not uniform (Figure S7 g). So we have optimized the synthesize time (30 min) which produces fully developed microspheres which constituted enhanced number density of nanosheets with more uniform size distribution.

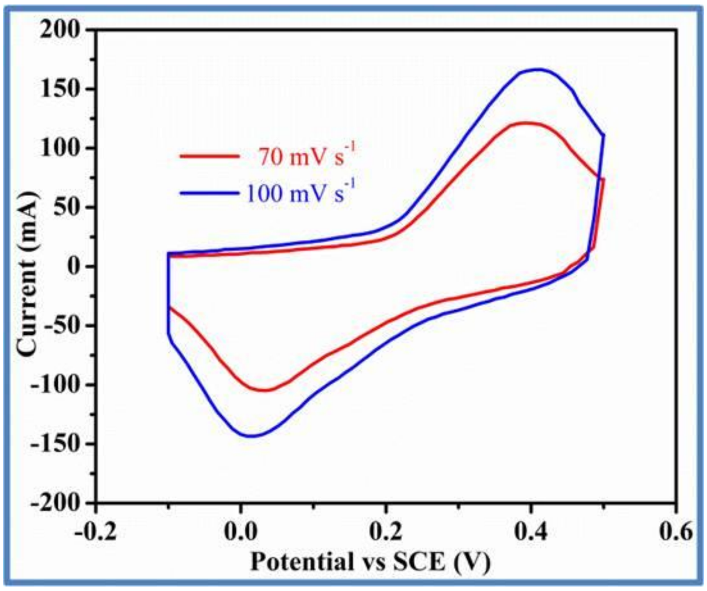

Figure S8. CV curves of porous $NiCo_2O_4$ microspheres at higher scan rate

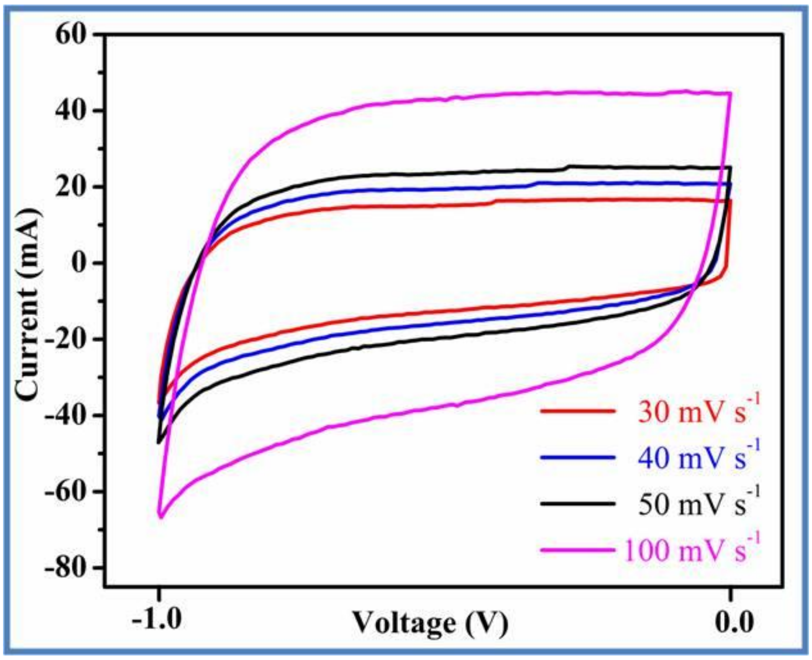

Figure S9. CV curves of active carbon at higher scan rate (30 to 100 mV s^{-1})

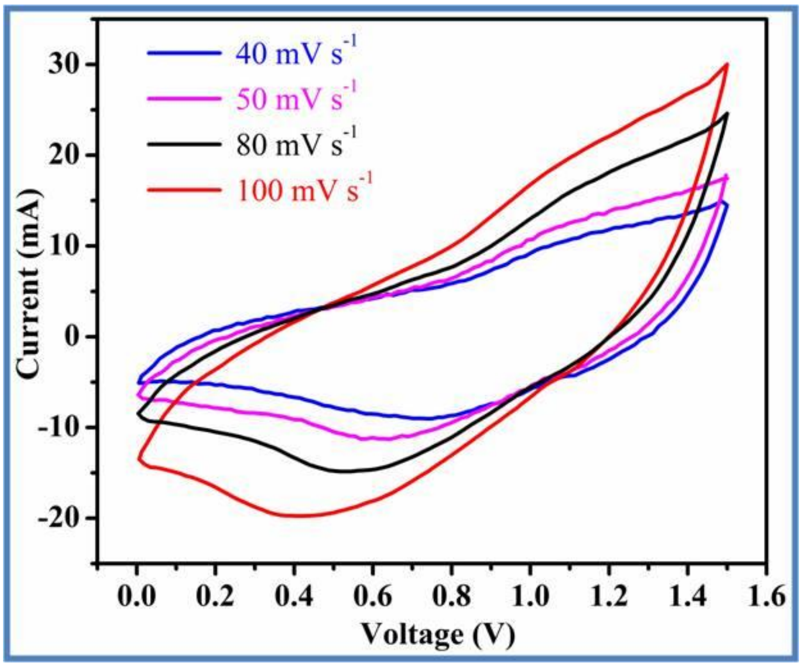

Figure S10. CV curves of asymmetric device at higher scan rate (40 to 100 mV s^{-1})

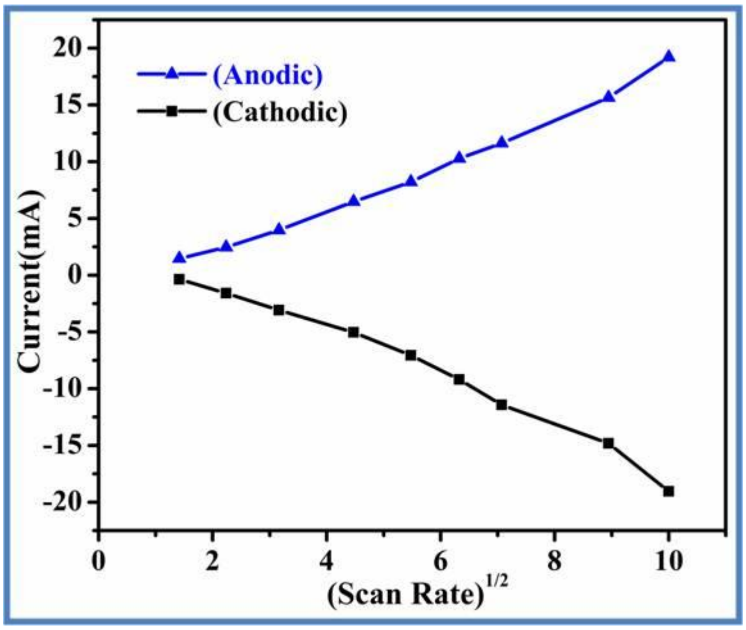

Figure S11. Oxidation current as a function of square root of scan rate of asymmetric device

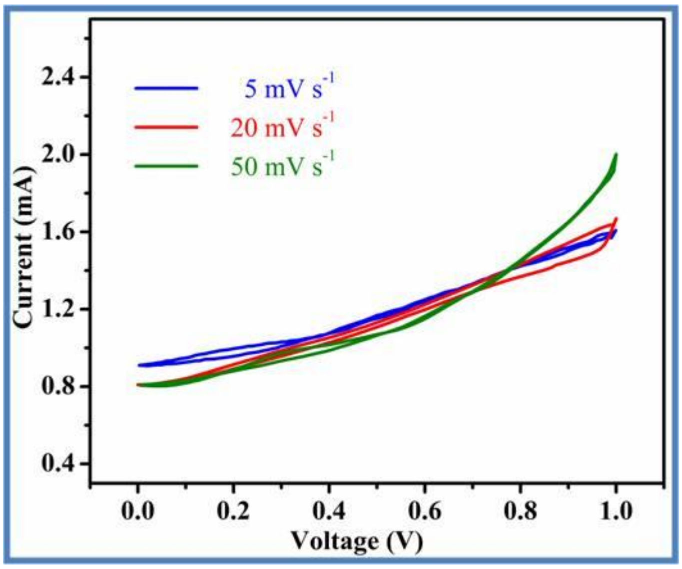

Figure S12. CV curves of device assembled of bare Ni foam at scan rate 5, 20 & 50 mV s^{-1}

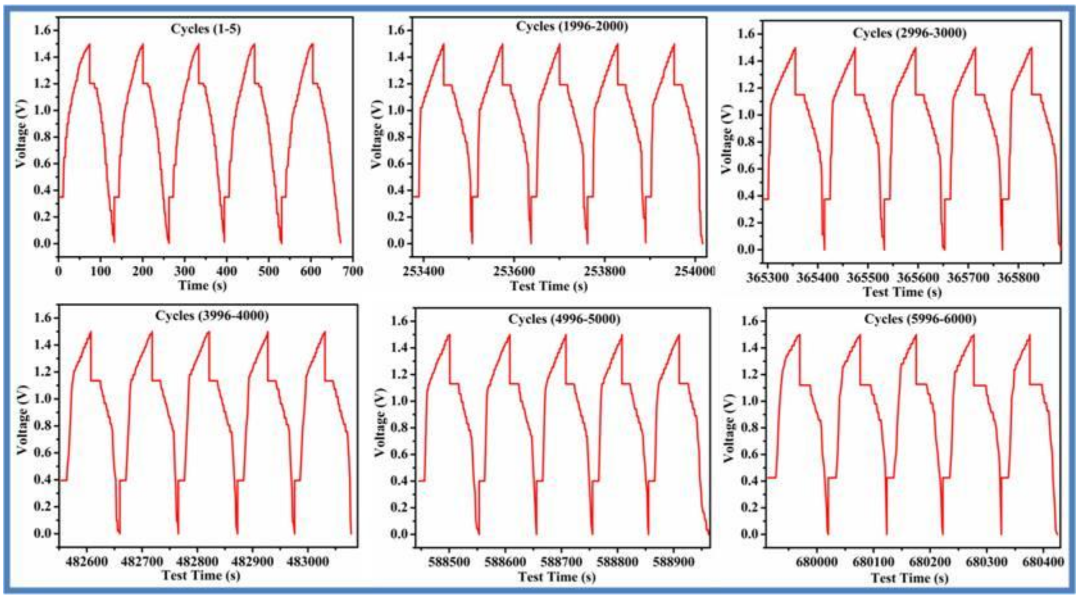

Figure S13. Galvanastic charge-discharge curves for few initial, intermediate & final cycles of asymmetric device at 6 mA cm^{-2}

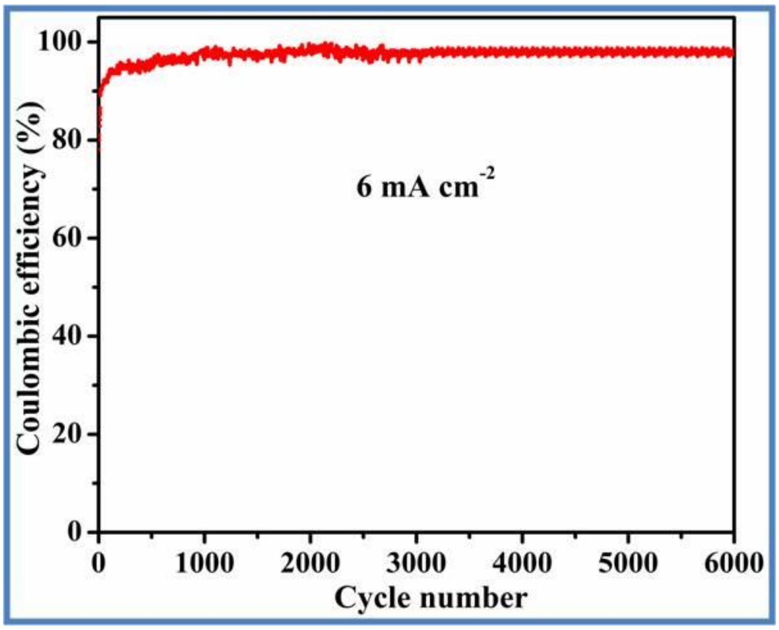

Figure S14. Coulombic efficiency of asymmetric device at 6 mA cm^{-2}

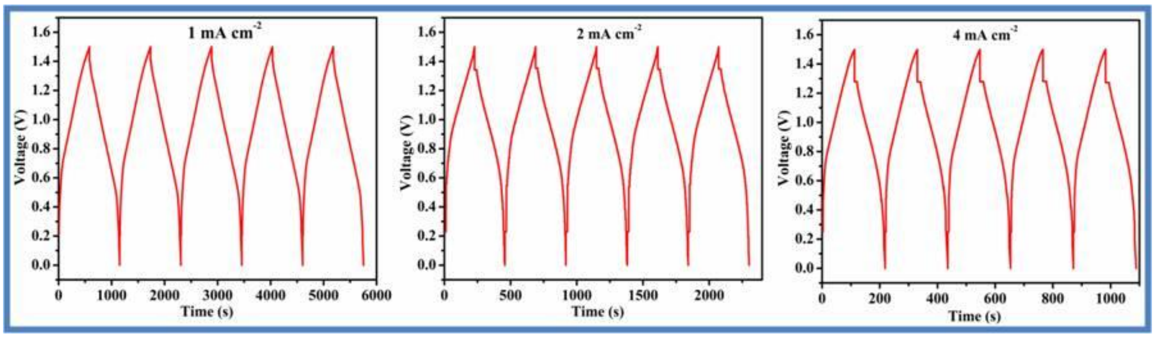

Figure S15. Galvanastic charge-discharge curves for few cycles of asymmetric device at 1, 2 & 4mA cm^{-2}

Table S2. Energy & maximum power densities of asymmetric device calculated from CD scans at different current densities

**Current density (mA cm^{-2})**	**Energy density (W h kg^{-1})**	**Max. power density (W Kg^{-1})**
1	19.1	1838.6
2	15.4	2265.6
4	14.2	3255
6	13.2	3628
8	11.7	4363
12	9.8	5400
16	9.1	5921

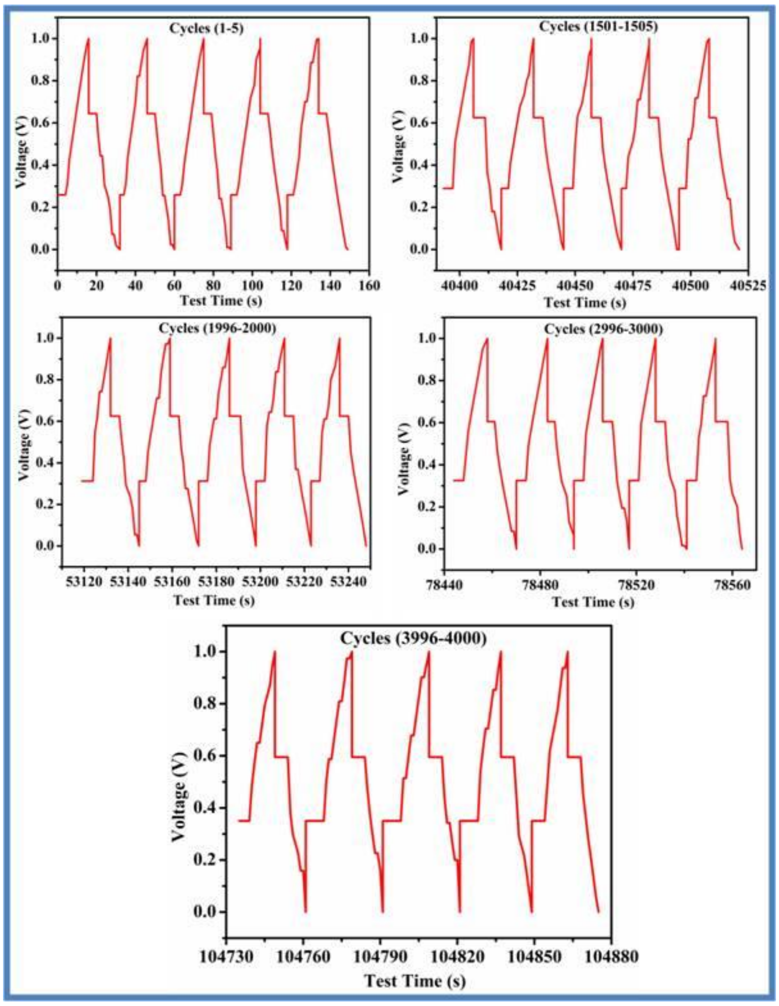

Figure S16. Galvanastic charge-discharge curves for few initial, intermediate & final cycles of symmetric device at 8 mA cm^{-2}

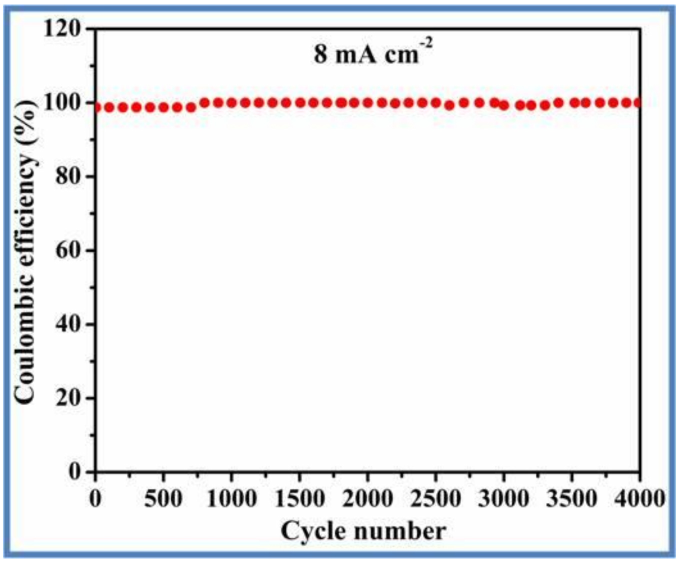

Figure S17. Coulombic efficiency of symmetric supercapacitor at 8 mA cm^{-2}

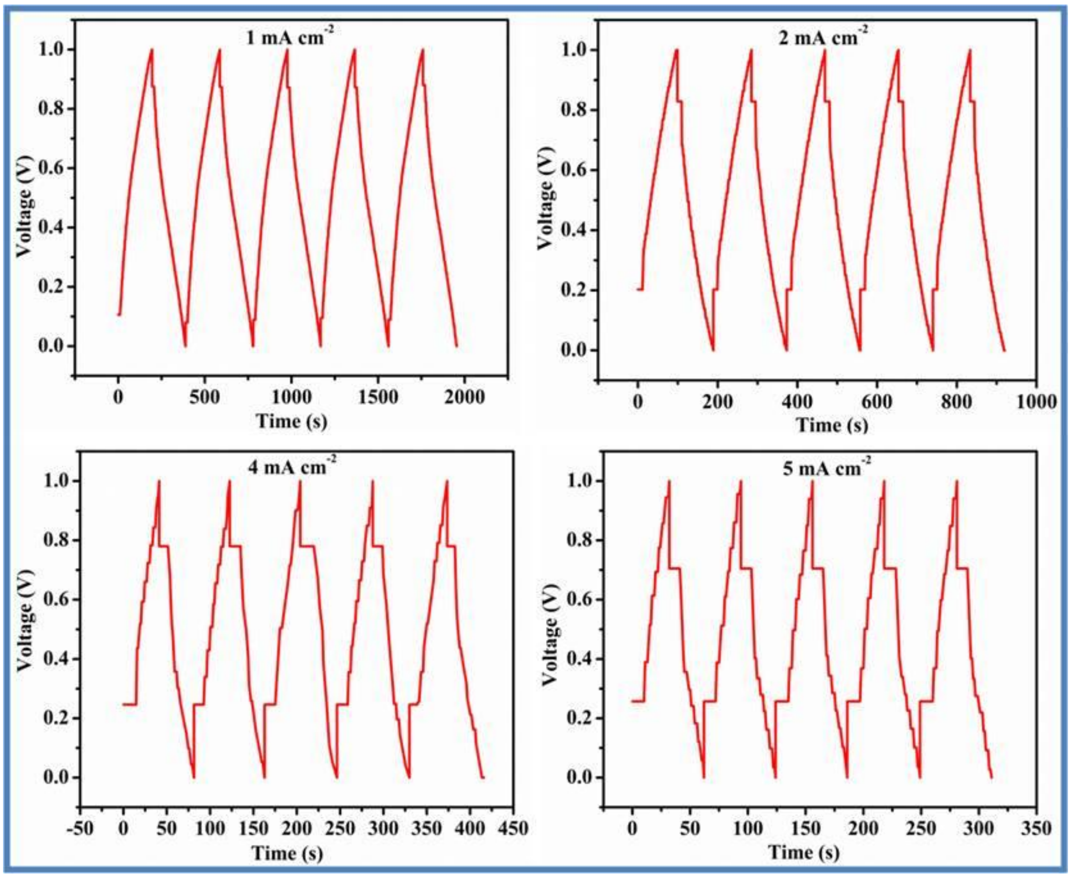

Figure S18. Galvanastic charge-discharge curves for few cycles of symmetric device at 1, 2, 4 & 5 mA cm^{-2}

Table S3. Energy & maximum power densities of symmetric device calculated from CD scans at different current densities

**Current density ($mA\ cm^{-2}$)**	**Energy density ($W\ h\ kg^{-1}$)**	**Max. power density ($W\ Kg^{-1}$)**
1	4.5	669.2
2	4.2	971.7
4	3.7	1514.9
5	3.5	1633.7
8	3.0	1872.4
12	2.5	2532.52

1. Stoller, M. D. & Ruoff, R. S. Best practice methods for determining an electrode material's performance for ultracapacitors. *Energ. Environ. Sci*. **3**, 1294-1301 (2010).
2. Makgopa, K. *et al.* A high-rate aqueous symmetric pseudocapacitor based on highly graphitized onion-like carbon/birnessite-type manganese oxide nanohybrids. *J. Mat. Chem. A* **3**, 3480-3490 (2015).
3. Feng, J.-X., Ye, S.-H., Lu, X.-F., Tong, Y.-X. & Li, G.-R. Asymmetric Paper Supercapacitor Based on Amorphous Porous Mn3O4 Negative Electrode and Ni(OH)2 Positive Electrode: A Novel and High-Performance Flexible Electrochemical Energy Storage Device. *ACS Appl. Mat. Interfaces* **7**, 11444-11451 (2015).
4. Luan, F. *et al.* High energy density asymmetric supercapacitors with a nickel oxide nanoflake cathode and a 3D reduced graphene oxide anode. *Nanoscale* **5**, 7984-7990 (2013).
5. Chen, P.-C., Shen, G., Shi, Y., Chen, H. & Zhou, C. Preparation and Characterization of Flexible Asymmetric Supercapacitors Based on Transition-Metal-Oxide Nanowire/Single-Walled Carbon Nanotube Hybrid Thin-Film Electrodes. *ACS Nano* **4**, 4403-4411 (2010).
6. Zhi, J. *et al.* Highly Conductive Ordered Mesoporous Carbon Based Electrodes Decorated by 3D Graphene and 1D Silver Nanowire for Flexible Supercapacitor. *Adv. Funct. Mater*. **24**, 2013-2019 (2014).
7. Xiang, C., Li, M., Zhi, M., Manivannan, A. & Wu, N. A reduced graphene oxide/Co3O4 composite for supercapacitor electrode. *J. Power Sources* **226**, 65-70 (2013).
8. Béguin, F., Presser, V., Balducci, A. & Frackowiak, E. Carbons and Electrolytes for Advanced Supercapacitors. *Adv. Mat.* **26**, 2219-2251(2014).
9. Qian, L., Gu, L., Yang, L., Yuan, H. & Xiao, D. Direct growth of NiCo2O4 nanostructures on conductive substrates with enhanced electrocatalytic activity and stability for methanol oxidation. *Nanoscale* **5**, 7388-7396 (2013).
10. Xiao, J. & Yang, S. Sequential crystallization of sea urchin-like bimetallic (Ni, Co) carbonate hydroxide and its morphology conserved conversion to porous NiCo2O4 spinel for pseudocapacitors. *RSC Adv.* **1**, 588-595 (2011).
11. Verma, S. *et al.* Nearly Monodispersed Multifunctional NiCo2O4 Spinel Nanoparticles: Magnetism, Infrared Transparency, and Radiofrequency Absorption. *J. Phy. Chem. C* **112**, 15106-15112 (2008).
12. Wang, X., Liu, W. S., Lu, X. & Lee, P. S. Dodecyl sulfate-induced fast faradic process in nickel cobalt oxide-reduced graphite oxide composite material and its application for asymmetric supercapacitor device. *J. Mat. Chem.* **22**, 23114-23119 (2012).
13. Kim, J. G., Pugmire, D. L., Battaglia, D. & Langell, M. A. Analysis of the NiCo2O4 spinel surface with Auger and X-ray photoelectron spectroscopy. *Appl. Surf. Sci*. **165**, 70-84 (2000).
14. Yuan, C. *et al.* Ultrathin Mesoporous NiCo2O4 Nanosheets Supported on Ni Foam as Advanced Electrodes for Supercapacitors. *Adv. Funct. Mater*. **22**, 4592-4597 (2012).
15. Roginskaya, Y. E. *et al.* Characterization of Bulk and Surface Composition of CoxNi1-xOy Mixed Oxides for Electrocatalysis. *Langmuir* **13**, 4621-4627 (1997).
16. Marco, J. F. *et al.* Characterization of the Nickel Cobaltite, NiCo2O4, Prepared by Several Methods: An XRD, XANES, EXAFS, and XPS Study. *J. Solid State Chem*.**153**, 74-81 (2000).
17. Thissen, A. *et al.* Photoelectron Spectroscopic Study of the Reaction of Li and Na with NiCo2O4. *Chem. Mater.* **17**, 5202-5208 (2005).
18. Lu, X.-F. *et al.* Hierarchical NiCo2O4 nanosheets@hollow microrod arrays for high-

performance asymmetric supercapacitors. *J. Mat. Chem. A* **2**, 4706-4713 (2014).
19. Yu, J., Wang, G., Cheng, B. & Zhou, M. Effects of hydrothermal temperature and time on the photocatalytic activity and microstructures of bimodal mesoporous TiO2 powders. *Appl. Catal. B-Environ.* **69**, 171-180 (2007).
20. Li, J., Xiong, S., Liu, Y., Ju, Z. & Qian, Y. High Electrochemical Performance of Monodisperse NiCo2O4 Mesoporous Microspheres as an Anode Material for Li-Ion Batteries. *ACS Appl. Mat. Interfaces* **5**, 981-988 (2013).
21. Zhang, G. Q., Wu, H. B., Hoster, H. E., Chan-Park, M. B. & Lou, X. W. Single-crystalline NiCo2O4 nanoneedle arrays grown on conductive substrates as binder-free electrodes for high-performance supercapacitors. *Energ. Environ. Sci*. **5**, 9453-9456 (2012).
22. Zhou, H., Li, D., Hibino, M. & Honma, I. A Self-Ordered, Crystalline–Glass, Mesoporous Nanocomposite for Use as a Lithium-Based Storage Device with Both High Power and High Energy Densities. *Angew. Chem. Int. Edit.* **44**, 797-802 (2005).
